# Response of iPSC-derived neurons from individuals with treatment-resistant depression to (2 *R*,6 *R*)-hydroxynorketamine and reelin: an exploratory study

**DOI:** 10.1038/s41398-025-03724-6

**Published:** 2025-11-18

**Authors:** Jenessa N. Johnston, Peixiong Yuan, Bashkim Kadriu, Nirmala Akula, Brandi Quintanilla, Shiyong Peng, Greg H. Jones, Anton Schulmann, Mani Yavi, Ioline D. Henter, Francis J. McMahon, Lisa E. Kalynchuk, Carlos A. Zarate, Hector J. Caruncho

**Affiliations:** 1 Experimental Therapeutics and Pathophysiology Branch, National Institute of Mental Health, National Institutes of Health, Bethesda, MD 20892 USA; 2 Section on the Genetic Basis of Mood and Anxiety Disorders, National Institute of Mental Health, National Institutes of Health, Bethesda, MD 20892 USA; 3 School of Medical Sciences, University of Victoria, Victoria, V8P 5C2 Canada

**Keywords:** Drug discovery, Stem cells, Depression

## Abstract

Treatment-resistant depression (TRD) is associated with worse clinical outcomes and longer course of illness. However, TRD is more difficult to model in animal phenotypes, suggesting that other experimental and translational models must be considered to properly address and research novel therapeutics. Reelin, an endogenous glycoprotein downregulated in depression, has shown rapid antidepressant-like effects akin to those of the N-methyl-D-aspartate receptor (NMDAR) antagonist ketamine. Interestingly, the antidepressant-like effects of both ketamine and reelin affect mechanistic target of rapamycin complex 1 (mTORC1) activity and that of its related downstream signalers. (2 *R*,6 *R*)-hydroxynorketamine (HNK) is a major metabolite of ketamine that, at therapeutic levels, appears to activate mTORC1 without antagonizing NMDARs. To model the effects of (2 *R*,6 *R*)-HNK and reelin on neurons from TRD participants, induced pluripotent stem cells (iPSCs) were reprogrammed from peripheral blood mononuclear cells collected from five females with TRD (mean=40.2 yrs) and then differentiated into cortical neurons. In iPSC-derived neurons from TRD participants, 50 nM reelin and 1 µM (2 *R*,6 *R*)-HNK had similar effects on the protein expression of GluA1, PSD-95, Dab1, Synapsin I, and p-ERK, with concentration-dependent increases observed at one hour that significantly decreased by 24 h. RNA sequencing revealed similar changes in gene expression between 50 nM reelin and 1 µM (2 *R*,6 *R*)-HNK at one hour, although only reelin upregulated mTORC1 signaling. While this work remains preliminary, the results suggest that iPSC-derived neurons could provide a valuable in vitro model to study TRD and hold promise for evaluating novel therapeutics such as (2 *R*,6 *R*)-HNK and reelin.

**Clinicaltrials.gov**: NCT02484456

## Introduction

Distinct limitations exist to using animal models for depression, particularly because these are often homogeneous, which is in direct contrast to the heterogeneity of the human experience of depression. Most current rodent models focus on testing response to standard antidepressants, but there is a noted lack of well-validated experimental and translational models specifically for treatment-resistant depression (TRD) [1, 2]. Although the definition varies, TRD is broadly defined by inadequate response to two or more classes of antidepressant regimens despite adequate dose, duration, and adherence to treatment. Individuals with TRD who do not respond to multiple classes of antidepressants, or show only a partial response, often face additional challenges, including functional impairment, lower quality of life, high relapse rates, and increased risk of suicidality—all core factors that are not well replicated by animal models [1–3]. In addition, the field’s inability to study brain tissue from living humans limits the molecular changes that can be observed in depression or in response to antidepressant treatments. In this context, a clear need exists to create translational models to help transition novel therapeutics from bench to bedside for the most treatment refractory patients.

The development of human inducible pluripotent stem cells (iPSCs) by reprogramming human fibroblasts, keratinocytes, and lymphocytes has allowed investigation into the cellular mechanisms of various disorders and therapeutics in humans [4, 5]. The complex polygenic landscape of depression makes iPSC models particularly valuable, as they capture the full genetic architecture of individual patients’ neurons—a capability previously unavailable to researchers beyond post-mortem samples. iPSC modeling has been successfully implemented to uncover key molecular insights across psychiatric disorders, including bipolar disorder, schizophrenia, autism spectrum disorders, and major depressive disorder (MDD) [6, 7]. To our knowledge, however, only two previous reports have examined iPSC modeling in individuals with TRD; both focused on the serotonergic system [8, 9].

The N-methyl-D-aspartate receptor (NMDAR) antagonist, glutamate modulator, and rapid-acting antidepressant ketamine is an effective treatment for TRD [10]. Although its precise mechanisms of action are varied and remain elusive, its primary therapeutic effects are thought to be mediated via activation of mechanistic target of rapamycin complex 1 (mTORC1) and related downstream signaling in the prefrontal cortex (PFC) and hippocampus [10, 11]. In addition, upregulation of proteins such as phosphorylated-mTOR (p-mTOR) and postsynaptic density-95 protein (PSD-95) as well as surface insertion of the glutamate A1 (GluA1) subunit of α-amino-3-hydroxy-5-methyl-4-isoxazolepropionic acid receptors (AMPARs) often accompanies ketamine’s antidepressant-like effects [12]. Despite its antidepressant efficacy, ketamine’s dissociative side effects and abuse potential limit its widespread use, underscoring the need to develop additional effective therapeutics for TRD.

In the context of developing novel treatments for TRD, one of ketamine’s major metabolites, (2 *R*,6 *R*)-hydroxynorketamine ((2 *R*,6 *R*)-HNK), has also shown promising rapid-acting antidepressant effects. Preclinical studies suggest that (2 *R*,6 *R*)-HNK retains antidepressant-like behavioral efficacy and exhibits strong synaptic potentiation effects at excitatory synapses while lacking the dissociative and abuse potential associated with ketamine and its enantiomers [13, 14]. A recent Phase I clinical trial found that (2 *R*,6 *R*)-HNK had a favourable safety and tolerability profile in healthy volunteers, with no evidence of anesthetic or dissociative effects. In addition, its pharmacokinetic profile revealed dose-proportional increases in exposure as well as increases in gamma power at low and medium doses [15]. Our laboratory recently initiated the first Phase II study to evaluate its efficacy in participants with TRD (NCT06511908).

The search for novel rapid-acting antidepressants has also led researchers to examine endogenous proteins that may share mechanistic overlap with the putative therapeutic effects of ketamine and (2 *R*,6 *R*)-HNK, and reelin has emerged as a promising candidate. Because reelin signaling may mediate some of the antidepressant effects of ketamine and (2 *R*,6 *R*)-HNK [16], exogenous reelin administration could help identify downstream therapeutic targets that could induce an antidepressant response. Reelin is a large extracellular matrix glycoprotein that is decreased in the hippocampus of individuals with mood disorders [17]. Previous research with in vitro and in vivo chronic stress animal models found that ketamine and reelin demonstrate similar effects (e.g., upregulation in long-term potentiation) [18–20], although reelin has not yet been investigated in individuals with TRD. Both reelin and ketamine have also been proposed as mediators of subchronic inflammation [21, 22], which is hypothesized to contribute to depressive symptomatology [23]. Reelin may also have dichotomous effects on inflammation; specifically, high levels of reelin expression within the blood may be pro-inflammatory and associated with disorders such as Alzheimer’s disease and multiple sclerosis [24], but reelin overexpression within the brain may have potential therapeutic effects in animal models for these disorders [22]. Studies also suggest that reelin increases synaptogenesis, synaptic potentiation, mTORC1 activation, and dendritogenesis, all of which have been implicated in ketamine’s antidepressant effects [25] and could thus prove effective at targeting TRD symptoms.

As noted above, molecular research into depression—including TRD—has been limited by our inability to directly investigate living *human* neurons. To address this gap, the current exploratory study sought to examine whether iPSC-derived neurons from participants with TRD, which model individual genetic variability, could be used to assess novel rapid-acting therapeutics in a translational manner. The impact of (2 *R*,6 *R*)-HNK and reelin on iPSC-derived neurons from participants with TRD was examined to determine molecular parallels between this novel in vitro method and previous in vivo research. The following proteins were measured: mTOR, extracellular-related signaling kinase (ERK), PSD-95, GluA1, Synapsin I, disabled adaptor protein (Dab1), tyrosine kinase B (TrkB), and NMDA-receptor subunit-2B (NR2B); all of these proteins are robustly implicated in the effects of rapid-acting antidepressants across multiple therapeutics [12, 26]. In addition, gene set enrichment analyses on bulk RNA-sequencing were conducted to determine changes in hallmark signaling pathways. The main objective of this series of experiments was to identify potential shared rapid antidepressant mechanisms between reelin and (2 *R*,6 *R*)-HNK. The study hypothesis was that broad changes would be observed after treatment with reelin and (2 *R*,6 *R*)-HNK in proteins and signaling pathways previously implicated in the pathophysiology of TRD and in antidepressant response to ketamine, including glutamatergic synaptic signaling and inflammatory responses.

## Materials and methods

### Collection of iPSCs

Peripheral blood mononuclear cells (PBMCs) were collected from five female TRD participants who were enrolled in a randomized clinical trial at the National Institute of Mental Health (NIMH) (NCT02484456); all participants provided written informed consent before taking part in the study, which was approved by the NIH Institutional Review Board. Participants were inpatients at the NIMH who met criteria for MDD without psychotic features, currently experiencing a depressive episode lasting at least four weeks; diagnosis was established using DSM-IV or DSM-5 criteria. Participants were also required to have a score of 18 or higher on the Hamilton Depression Rating Scale (HAM-D) and a history of inadequate response to at least one conventional antidepressant treatment, operationally defined using the modified Antidepressant Treatment History Form (ATHF). Power analyses, conducted as based on criteria laid out by Brunner and colleagues [27] for an experimental design including multiple isogenic pairs, determined that a minimum of four samples would be needed to properly assess treatment differences. All five participants selected for iPSC generation had not responded to at least three antidepressant treatments (mean=6.7, SD = 4.2). In addition, one participant had previously failed to respond to bilateral electroconvulsive therapy (ECT), and two participants had failed to respond to lithium. Participant demographics can be found in Table 1, and further information on lifetime failed antidepressant trials can be found in Table 2.

**Table 1:** Participant Demographics.

	Treatment-Resistant Depression (n = 5)
**Age**	
Mean (SD)	40.2 (8.6)
Range	31 - 47
**Sex ((n,%))**	
Female	5 (100)
Male	0 (0)
**Race ((n,%))**	
White	3 (60)
Black or African American	2 (40)
Other	0 (0)
**Baseline MADRS**	
Mean (SD)	30.7 (3.1)
Range	29 - 38
**Number of Failed Antidepressant Trials**	
Mean (SD)	6.7 (4.2)
Range	3 - 13
**Age of Onset**	
Mean (SD)	15.5 (4.3)
Range	10 - 23

*MADRS* montgomery-asberg depression rating scale.

**Table 2:** Failed Medication Trials for Participants with TRD (n = 5).

		Lifetime Total - Failed Medication Trials at Adequate Dose and Duration					
	Overall duration of illness (years)	SSRI trials	SNRI trials	TCA trials	MAOI trials	Other antidepressant trials	Augmentation trials
Mean	24.3	2.3	1.2	1.0	0.3	1.7	3.8
Standard Deviation	16.4	2.2	1.0	1.3	0.5	0.8	3.3
Range	6 - 49	0 - 5	0 - 3	0 - 3	0 - 1	0 - 3	0 - 7

*TRD* treatment-resistant depression, *SSRI* selective serotonin reuptake inhibitor, *SNRI* serotonin-norepinephrine reuptake inhibitor, *TCA* tricyclic antidepressant, *MAOI* monoamine oxidase inhibitor.

Generation of human iPSCs from PBMCs was conducted at the National Heart, Lung, and Blood Institute (NHLBI/NIH) iPSC Core Facility using non-integration methods. The STEMdiff^TM^ embryoid body protocol was followed for 19 days to develop single-cell neural progenitor cells (NPCs). Neural induction was confirmed through visual inspection, and neural rosettes were selected. Cells were seeded onto PLO/laminin-coated dishes (half with poly-L-lysine coated coverslips) at a density of 1.5 ×10^4 – 3 ×10^4 cells/cm^2^. The cells were then incubated at 37 °C with 5% circulating CO_2_ and supplemented with BrainPhys^TM^ Neuronal Medium, 1% N2 Supplement-A, 2% Neurocult^TM^ SM1 Neuronal Supplement, 20 ng/ml glial cell line-derived neurotrophic factor (GDNF), 20 ng/ml brain-derived neurotrophic factor (BDNF), 1 mM Dibutyryl-cyclic adenosine monophosphate (cAMP), and 200 nM ascorbic acid for 10 weeks with half-medium changes every two days. Cells were checked for mycoplasma contamination using the MycoAlert® Mycoplasma Detection Kit (Lonza Bioscience, Rockville, MD), and a CCK-8 cell counting kit (Millipore Sigma, St. Louis, MO) was used as a viability assay. Neuronal maturation was verified through morphological immunocytochemical (ICC) analysis of MAP2 (ab32454, Abcam) and PSD-95 (7E3-1B8, Invitrogen). Briefly, after fixation, cells for ICC analyses were permeabilized by incubating in 0.5% Triton X-100 in PBS for five minutes then incubating overnight in primary antibodies (PSD-95, MAP2) after blocking. Fluorescent images were taken at 60X magnification on a Nikon A1R confocal microscope.

### Treatment of Cultures

After verification of a maturate phenotype at 10 weeks [28], cells were divided into five different conditions: 1) vehicle + DMSO; 2) 5 nM reelin; 3) 10 nM reelin; 4) 50 nM reelin; or 5) 1 μM (2 *R*,6 *R*)-HNK. Concentrations of reelin were chosen based on previous in vitro work conducted in parallel with effective antidepressant-like doses in chronic stress animal models [19, 29], and the concentration of (2 *R*,6 *R*)-HNK was chosen based on previous research demonstrating efficacy in iPSC-derived neurons from healthy volunteers [30]. The effects of all doses were assessed at both one hour and 24 h post-administration to measure the short and long-term effects of reelin and (2 *R*,6 *R*)-HNK. Each cell line was cultured in enough wells to allow for multiples of every treatment concentration; there were 40 wells per cell line to allow for eight biological replicates in all treatment analyses, with no wells pooled together for downstream analyses. Wells were rinsed and scraped for Western blot and RNA sequencing analyses.

### Western Blot

For Western Blot analysis, cells were homogenized in M-PER mammalian protein extraction reagent (Thermo Scientific, Waltham, MA. Cat#78501) supplied with protease and phosphatase inhibitor cocktail (Thermo Scientific, Cat#78442). A total of 10 μg of protein was electrophoretically resolved in 4–12% NuPAGE Bis-Tris gels (Invitrogen/Thermo Scientific) then transferred onto 0.2 µm nitrocellulose membranes via a semi-dry transfer method in the Trans-Blot Turbo Transfer System (Bio-Rad, Hercules, CA). Membranes were blocked using 5% (w/v) milk for unphosphorylated proteins and 5% (w/v) bovine serum albumin (BSA) for phosphorylated proteins for one hour at room temperature. To ensure proper phosphorylation ratio measurements, the same membranes were used to quantify both total protein and phosphorylated protein for mTOR (#2972S, CST) and ERK (#5376S, CST). Other proteins analyzed were PSD-95 (#2507S, CST), Synapsin I (#6710, CST), GluA1 (#13185S, CST), Dab1 (#3328S, CST), TrkB (#4603S, CST), and NR2B (UC Davis). Membranes were stripped with Restore™ PLUS Western Blot Stripping Buffer (Thermo Scientific, Cat#46430). ECL images were captured with Syngene G:Box Imaging system. Bend densitometry was quantified with Image J. These proteins have repeatedly been shown to be increased in glutamatergic synaptic signaling and in response to rapid-acting antidepressants [10, 11] and were chosen to determine the in vitro impact that putative rapid-acting antidepressants would have in patient-derived cell lines. Further confirmation of the proteomic analyses was conducted via immunocytochemical analyses (see Supplement).

### RNA Sequencing

The predominant goal of this series of experiments was to characterize potentially shared rapid antidepressant mechanisms between reelin and (2 *R*,6 *R*)-HNK. The timepoint of one-hour post-treatment was chosen because significant clinical response to ketamine has been shown to occur within that timeframe, which would allow characterization of the effects of multiple reelin concentrations with a limited sample size. RNA was extracted using the Qiagen RNeasy Kit for bulk RNA experiments. RNA purity was verified using a NanoDrop spectrophotometer (Thermo Scientific, Waltham, MA). The purified RNA samples were then frozen and sent to Novogene (Sacramento, CA) for processing. After standard quality control procedures, FastQ files were downloaded and aligned to the Ensembl GRCh38.p14 human genome assembly. Gene expression was quantified by aligning reads to the hg38 genome using STAR default parameters, and raw counts were quantified with featureCounts [31, 32]. Genes below a minimum threshold of 0.1 counts-per-million in at least five samples, genes on the Y chromosome, and genes of ribosomal or mitochondrial origin were filtered prior to normalization and downstream analysis.

### Statistics

Statistics for the Western blot results were conducted using SPSS (v20.0, IBM). To determine the impact of reelin and (2 *R*,6 *R*)-HNK on iPSCs, the Kruskal-Wallis test was used to correct for non-parametric data. Dunn’s multiple comparison test was used for post-hoc comparisons. For bulk RNA-sequencing analysis, treatment and time effects were assessed while controlling for individual cell line variability. Significant variance contributors, as identified by VariancePartition [33], were also included as random effects. After removing genes expressed at low frequency, differentially expressed gene (DEG) analysis was performed using Dream, a modified version of limma/voom analysis designed to assess drug-treatment effects [34]. Dream is a linear mixed modeling process that accounts for powerful within-subjects testing such as in iPSC-derived cell lines where each participant is treated with all drug conditions. Hallmark gene sets from the bulk RNA sequencing dataset were annotated through Zenith [34] and adjusted for false discovery rate (FDR).

## Results

To determine the effects of putative rapid-acting antidepressants, reelin and (2 *R*,6 *R*)-HNK were applied to mature iPSC-derived cortical neurons from participants with TRD (n = 5). After one and 24 h of treatment, cells were collected to assess time-dependent changes in synaptic protein and gene expression.

### Immunocytochemistry verified a mature neuronal phenotype within cultures

**Fig. 1:**
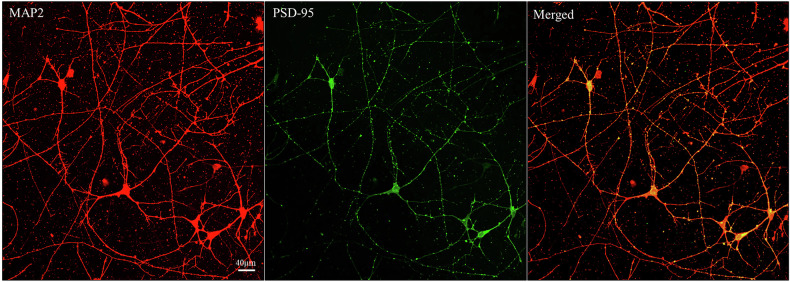
Immunofluorescent verification of neuronal morphology. Immunocytochemical analyses revealed widespread expression of MAP2 within neurons and postsynaptic density-95 protein (PSD-95) primarily localizing with dendrites, suggesting a mature neuronal phenotype. Images were collected at 60X magnification on a Nikon A1R confocal microscope.

After 10 weeks of maturation, immunofluorescent staining of MAP2 and PSD-95 confirmed a mature neuronal phenotype within our iPSC-derived cultures (Fig. 1). The experiments were thus continued to determine time- and drug-dependent effects of reelin and (2 *R*,6 *R*)-HNK on cortical neurons from individuals with TRD. Viability was confirmed using the CCK-8 assay, showing no significant toxicity at the concentrations used in the main experiments.

### Western Blotting revealed time-dependent changes in synaptic-strength related proteins

As previously mentioned, the hypothesized overlap in the mechanisms of action of reelin and (2 *R*,6 *R*)-HNK lie within the synapse. Changes in proteins related to mTORC1 activation, synaptic strength (e.g., PSD-95), and excitatory signaling (e.g., GluA1) were all assessed through a Kruskal-Wallis test to determine if these putative rapid-acting antidepressants would have similar synaptic-level effects within iPSC-derived cortical neurons.

**Fig. 2:**
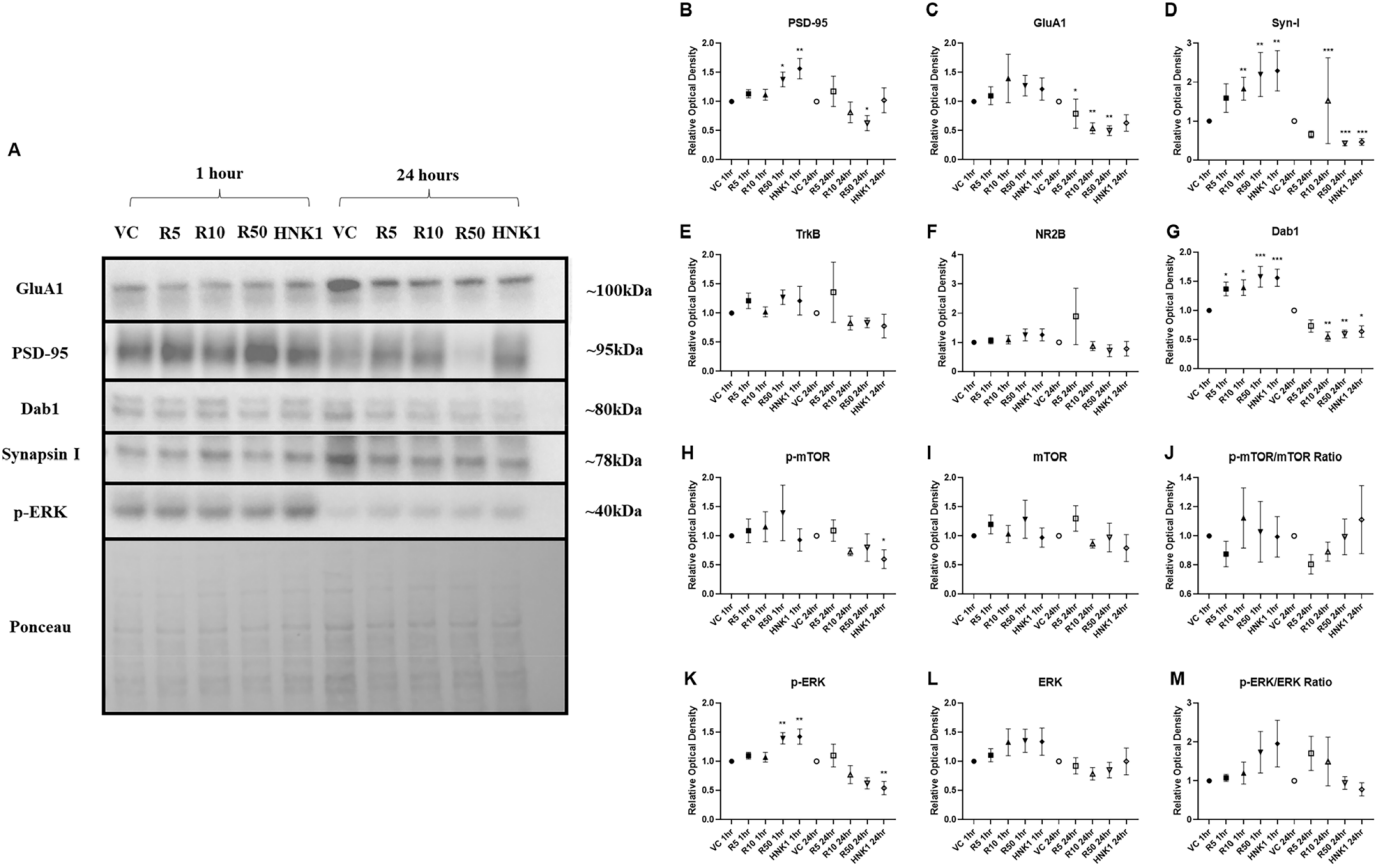
The impact of reelin and (2 *R*,6 *R*)-hydroxynorketamine (HNK) on induced pluripotent stem cell (iPSC)-derived neurons from individuals with treatment-resistant depression (TRD). **A** Representative Western Blot images of the impact of reelin and (2 *R*,6 *R*)-HNK on iPSC-derived neurons from TRD participants at 1 h and 24 h. All proteins were normalized to Ponceau (total protein expression). Images shown for proteins with significant differences in expression. **B-M** Graphs showing expression levels of post-synaptic density (PSD)-95, GluA1, Synapsin I, tyrosine kinase B (TrkB), NR2B, disabled adaptor protein 1 (Dab1), mechanistic target of rapamycin (mTOR), phosphorylated mTOR (p-mTOR), ratio of mTOR activity, extracellular-regulated signaling kinase (ERK), p-ERK, and ratio of ERK activity after treatment. VC, vehicle control; R5, reelin 5 nM; R10, reelin 10 nM; R50, reelin 50 nM; HNK1, (2 *R*,6 *R*)-HNK 1 µM; *p < 0.05, **p < 0.01, ***p < 0.001 vs. VC.

Both reelin and (2 *R*,6 *R*)-HNK demonstrated similar effects at one hour and 24 h post-administration in iPSC-derived neurons from TRD participants (Fig. 2). PSD-95 showed significant changes after both one (*H*(4) = 13.68, *p* = 0.0078) and 24 h of treatment (*H*(4) = 9.979, *p* = 0.0408). Dunn’s post-hoc tests found that PSD-95 was significantly increased from the one-hour vehicle control with 50 nM of reelin (*p* = 0.032) and 1 µM of (2 *R*,6 *R*)-HNK (*p* = 0.0021) at one hour. In contrast, PSD-95 was downregulated after 24 h only with 50 nM of reelin (*p* = 0.0162).

Synapsin I showed concentration-dependent changes at both one hour (*H*(4) = 17.51, *p* = 0.0015) and 24 h (*H*(4) = 22.98, *p* = 0.0001), with post-hoc tests revealing consistent upregulation of expression at one hour compared to relative vehicle control (reelin 10 nM, *p* = 0.0064; reelin 50 nM, *p* = 0.0016; (2 *R*,6 *R*)-HNK 1 µM, *p* = 0.0032) and consistent downregulation at 24 h (reelin 10 nM, *p* = 0.0009; reelin 50 nM, *p* = 0.0002; (2 *R*,6 *R*)-HNK 1 µM, *p* = 0.0004).

All treatments increased levels of Dab1, an intracellular adaptor protein essential for reelin’s effects, compared to vehicle control-treated cell lines after one hour (*H*(4) = 0.0011, *p* = 0.0011; reelin 5 nM, *p* = 0.0331; reelin 10 nM, *p* = 0.032; reelin 50 nM, *p* = 0.0009; (2 *R*,6 *R*)-HNK 1 µM, *p* = 0.0009), which was paralleled by decreased expression of Dab1 at 24 h compared to control (*H*(4) = 16.55, *p* = 0.0024; reelin 10 nM, *p* = 0.0014; reelin 50 nM, *p* = 0.0037; (2 *R*,6 *R*)-HNK 1 µM, *p* = 0.0217).

Finally, while GluA1 was not upregulated in response to any of the five treatments at one hour, the AMPAR subunit was significantly downregulated at 24 h (*H*(4) = 15.42, *p* = 0.0039) in response to all concentrations of reelin (5 nM, *p* = 0.0311; 10 nM, *p* = 0.0068; 50 nM, *p* = 0.0015). No significant results were found in expression of TrkB or NR2B.

Mixed results were seen with regard to activity indicators of the major cellular signaling pathways mTOR and ERK. No significant differences were observed at either timepoint for p-mTOR, mTOR, or the ratio of mTOR activity except for a slight downregulation of p-mTOR at 24 h with (2 *R*,6 *R*)-HNK (*H*(4) = 11.77, *p* = 0.0191; (2 *R*,6 *R*)-HNK 1 µM, *p* = 0.0197). However, upregulation of p-ERK was noted at one hour (*H*(4) = 19.51, *p* = 0.0006; reelin 50 nM, *p* = 0.0016; (2 *R*,6 *R*)-HNK 1 µM, *p* = 0.0014); it was also significantly decreased (*H*(4) = 13.35, *p* = 0.0097) with (2 *R*,6 *R*)-HNK treatment at 24 h (*p* = 0.0072). No significant differences were found in expression of total ERK or in the ratio of ERK activity.

### RNA sequencing showed a concentration-specific overlap of reelin and (2 R,6 R)-HNK

**Fig. 3:**
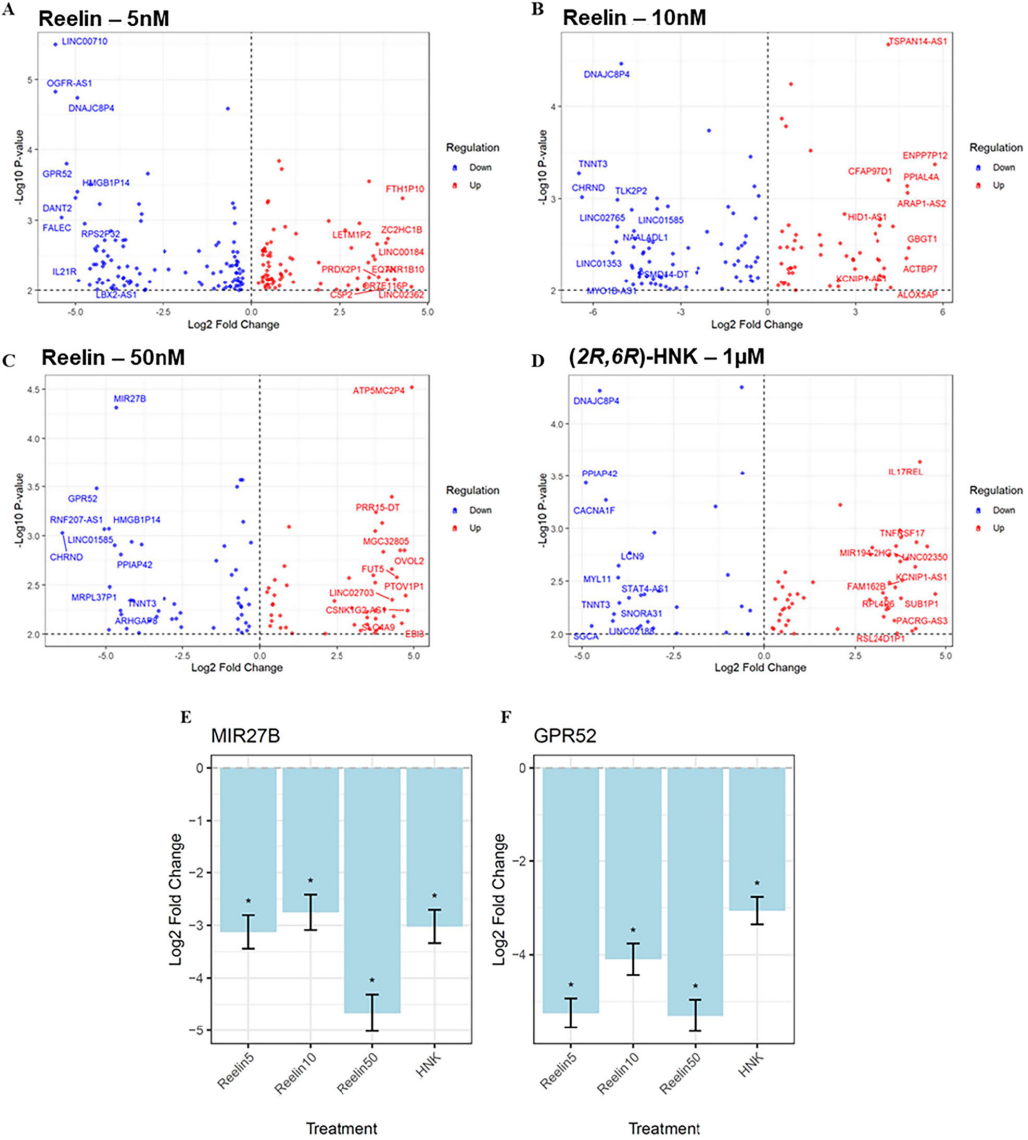
Significant differentially expressed genes (DEGs) one hour after treatment. **A-D** Log-fold change of significant DEGs from vehicle-treated neurons controlling for cell line. Blue indicates downregulation from vehicle control, and red indicates upregulation from vehicle control. Threshold was set at uncorrected p < 0.01. **E**, **F**: Log-fold change of significant overlapping genes from all treatments. *False discovery rate (FDR)-adjusted p < 0.05 from vehicle control.

One hour after treatment, the iPSC-derived neurons were assessed for changes in gene expression patterns. Limited sample availability from the 24-h cohort prevented RNA-sequencing analyses of this later timepoint. Differential gene expression analysis of bulk RNA sequencing data was conducted with Dream. Due to the exploratory nature of the study and the small sample size (n = 5), the threshold for differential expression was set at nominal p < 0.01. DEGs were found across all four treatment groups (reelin 5 nM: 194; reelin 10 nM: 133; reelin 50 nM: 98; (2 *R*,6 *R*)-HNK: 81) (Fig. 3A-D; Supplemental Tables S2-S5). Three common DEGs were identified across all treatments: *miR-27b*, *GPR52*, and *DNAJC8P4*, a pseudogene of the DnaJ heat shock protein family. *miR-27b* and *GPR52* were both significantly downregulated across all treatments compared to vehicle control (p < 0.01) (Fig. 3E-F).

**Fig. 4:**
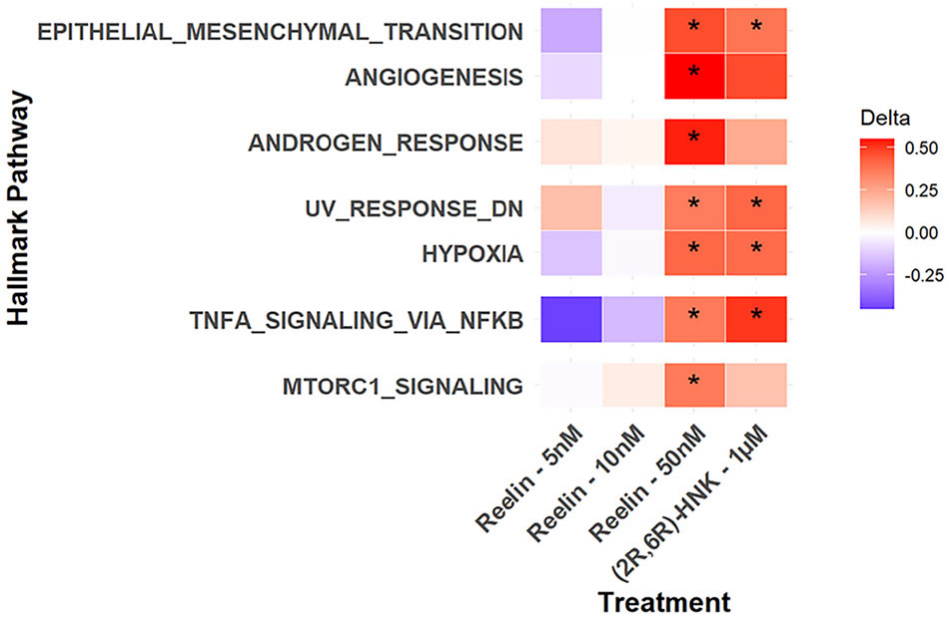
Significant hallmark pathway regulation one hour after treatment. Delta values indicate direction of change from vehicle treatment, with blue indicating downregulation and red indicating upregulation. At 50 nM, reelin significantly upregulated all pathways displayed. (2 *R*,6 *R*)-hydroxynorketamine (HNK) also significantly upregulated epithelial to mesenchymal transition (EMT), hypoxia, and tumor necrosis factor (TNF-α) signaling, and inhibited UV response. No other significant changes in pathways were observed across treatment groups. Bolded values indicate significance, with * p < 0.05.

While the two lower concentrations of reelin had no significant impact on signaling pathways, the highest concentration of reelin (50 nM) significantly increased six MSigDB human hallmark signaling pathways identified through Zenith (Fig. 4): epithelial-mesenchymal transition (EMT) (FDR = 0.017; δ = 0.47), androgen response (FDR = 0.017; δ = 0.53), hypoxia (FDR = 0.028; δ = 0.41), mTORC1 signaling (FDR = 0.042; δ = 0.37), tumor necrosis alpha (TNF-α) signaling via nuclear factor kappa-light-chain-enhancer of activated B cells (NF-κβ) (FDR = 0.042; δ = 0.37), and angiogenesis (FDR = 0.049; δ = 0.47), and it downregulated UV response (FDR = 0.049; δ = 0.41). Echoing the Western Blot results, (2 *R*,6 *R*)-HNK behaved most similarly to the highest concentration of reelin (50 nM), significantly increasing three overlapping pathways: EMT (FDR = 0.034; δ = 0.38), hypoxia (FDR = 0.034; δ = 0.40), TNF-α signaling via NF-κβ (FDR = 0.013; δ = 0.51) and downregulating UV response (FDR = 0.034; δ = 0.41). No other hallmark pathways were significantly changed after treatment.

**Fig. 5:**
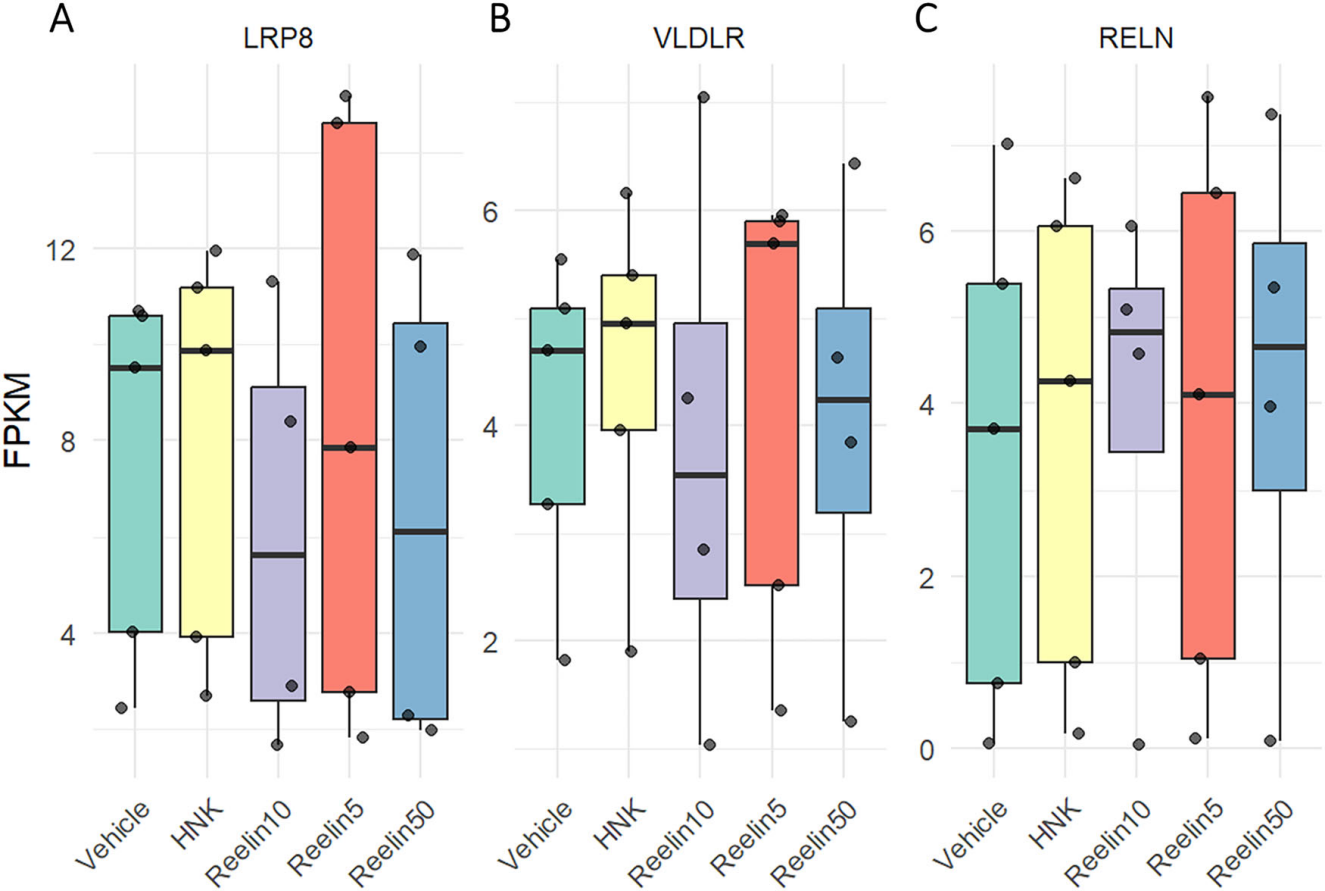
FPKM expression of reelin and reelin receptors. **A**, **B** Fragments Per Kilobase of transcript per Million (FPKM) expression of *LRP8* (otherwise known as Apolipoprotein E Receptor 2) and Very Low-Density Lipoprotein Receptor (*VLDLR*), reelin’s canonical receptors. No significant differences in expression were found at one hour across all treatment groups. **C** *RELN* expression did not significantly change across all treatment groups.

To determine whether changes in downstream signaling pathways were due to increased expression of reelin’s canonical receptors, Apolipoprotein E receptor 2 (ApoER2, or *LRP8*) and Very Low-Density Lipoprotein Receptor (*VLDLR*) were assessed within the bulk RNA-sequencing data (Figs. 5A, 5B). No significant differences were found across any treatment groups, though the high expression levels of each receptor support reelin’s efficacy within these cell cultures. *RELN* expression was also assessed (Fig. 5C) to verify the potential presence of endogenous reelin.

## Discussion

This exploratory study investigated the effects of 1µᴍ (2 *R*,6 *R*)-HNK and varying concentrations of reelin on iPSC-derived neurons obtained from individuals with TRD. The parallels found in our iPSC-derived neurons, animal models, and clinical research demonstrate that iPSC-derived neurons can not only provide insights into polygenic psychiatric illnesses and the mechanisms of established treatments but also serve as an in vitro platform for screening, translating, and eventually personalizing promising novel therapeutics. Using iPSC-derived neurons from different patient populations allows for the unique opportunity to research changes in human neuronal tissue after exposure to established and novel therapeutics.

Notably, reelin had time- and concentration-dependent impacts that paralleled the effects of (2 *R*,6 *R*)-HNK on cell lines derived from participants with TRD. Proteins integral to synaptic plasticity such as PSD-95, Synapsin I, Dab1, and p-ERK were all significantly increased at one hour in response to both (2 *R*,6 *R*)-HNK and higher concentrations of reelin, underscoring that the two treatments had similar effects. These effects were reversed at the 24-h mark, suggesting either compensatory downregulation after induction of structural plasticity or that prolonged exposure to reelin or (2 *R*,6 *R*)-HNK might have deleterious effects on cell cultures. In addition, the increase in Synapsin I suggests that both reelin and (2 *R*,6 *R*)-HNK have effects at the pre-synaptic level and may increase neurotransmitter release, which supports previous research on ketamine’s pre-synaptic mechanisms [35]. p-ERK expression was upregulated by both agents at the one-hour timepoint, echoing previous work demonstrating its rescue by ketamine in an animal model of depression [36]. Previous research also found that ERK was downregulated in the PFC of individuals with MDD [37] and noted a correlation between ketamine’s antidepressant effects and ERK signaling [38]. Recent research has implicated cAMP signaling in the antidepressant actions of ketamine and (2 *R*,6 *R*)-HNK [39, 40]. cAMP signaling is a known mediator of ERK signaling [41] and may be a key driver of these antidepressant-like responses. Future research should determine whether cAMP signaling is necessary for the observed effects of reelin and (2 *R*,6 *R*)-HNK within this model. Interestingly, immunocytochemical analyses (see Supplement) revealed time- and dose-dependent increases in proxy TrkB and NR2B expression, paralleling Western blot results; the highest levels of expression were found at one hour after treatment with 50nᴍ of reelin and (2 *R*,6 *R*)-HNK. However, further research is needed to establish the most appropriate methods for ICC analysis. These findings, which used punctal analyses as a proxy for protein expression, should be interpreted with caution.

Given the similarities between the molecular actions of high-dose reelin and (2 *R*,6 *R*)-HNK, this study also assessed common differentially expressed genes in all treatment groups; results were particularly interesting for *miR-27b* and *GPR52*. *miR-27b* was previously found to be impacted by chronic stress exposure in a rodent model [42] and has been associated with reward-related brain activation [43]. Clinically, plasma concentrations of *miR-27b* were found to correlate with the efficacy of alprazolam in patients with comorbid anxiety and alcohol use disorders [44]. The decrease in *GPR52* expression observed in this study after treatment with all doses of reelin and (2 *R*,6 *R*)-HNK further supports the role of therapeutically targeting G protein-coupled receptors in MDD and TRD [45]. *GPR52* encodes an orphan Gs-coupled receptor that is highly expressed in the brain, particularly in striatal regions where it colocalizes with D2 dopamine receptors [46]. This finding aligns with emerging evidence that GPR52-dependent regulation of the nucleus accumbens-ventral pallidum-ventral tegmental area circuit may be crucial for maintaining proper reward processing and motivation [47]. In addition, *GPR52* knockout mice displayed reduced anxiety-like symptoms [48], and a *GPR52* inverse agonist was recently found to ameliorate chronic stress-induced deficits in dopaminergic activity and reward motivation [47]. An orally available *GPR52* agonist (HTL0048149) has recently advanced into Phase I human clinical studies with future potential in schizophrenia [49], providing direct translational potential for this marker. Finally, *ARHGAP8*, a Rho GTPase involved in synaptic structure and AMPAR-mediated transmission, was found to be decreased with the highest concentration of reelin. *ARHGAP8* increases have previously been linked to neurodevelopmental and neuropsychiatric disorders [50–52], and decreasing its expression may be another method by which reelin exerts antidepressant-like effects.

It is also interesting to note that the global metabolic regulation through ERK observed here aligns with our RNA sequencing findings of altered mTORC1 signaling, as both pathways are known to coordinate cellular energy status and growth signaling. Here, mTORC1-related signaling was significantly upregulated by both (2 *R*,6 *R*)-HNK and reelin (50 nM) one hour after treatment exposure, consistent with previous reports [14]. While mTOR signaling dynamics can be challenging to capture via Western Blot, the transcriptional signature of this pathway across our RNA sequencing data underscores that these multiple complementary methodologies detected clinically relevant signaling. Interestingly, upregulation of the androgen response was also observed after treatment with 50 nM of reelin.

This study also found that the highest concentration of reelin (50 nM) significantly increased TNF-α signaling via NF-κβ. Though generally considered pro-inflammatory, increases in TNF-α signaling are important for neuronal plasticity, cognition, and CNS development [53]. TNF-α plays a vital role in synaptic scaling and plasticity by modulating both excitatory and inhibitory transmission via regulation of AMPARs and gamma aminobutyric acid (GABA) receptors [54, 55]. In MDD, chronic upregulation of TNF-α has been associated with worsened symptomatology and poorer clinical outcomes [56]. However, animal models found transient increases in TNF-α signaling after acute administration of ketamine that were associated with antidepressant-like effects [57], which parallels changes observed in our RNA sequencing results. The exact role of reelin in mediating inflammatory response, particularly within the brain, is still under discussion. High levels of circulating reelin have been shown to promote leukocyte infiltration and thrombosis as well as positively correlate with increased concentrations of cytokines [24, 58]. While high levels of reelin have been associated with inflammatory conditions, homeostatic balance may be necessary for proper neuronal functioning of reelin. Mice with reduced reelin signaling are more susceptible to chronic stress-induced behavioral and biological deficits, and exogenous reelin supplementation was found to rescue these deficits [59, 60]. As with ketamine, a transient increase in inflammation could be beneficial to rapid-acting antidepressant response. Future research should ascertain whether similar responses are found within cell lines derived from healthy volunteers.

Several reviews have discussed both the limitations and merits of iPSC modeling for neuropsychiatric diseases, emphasizing the need for thorough model characterization [4–6]. Modeling depression using iPSCs presents unique challenges due to the moderate genetic heritability of the disorder (~30 - 50%) as well as its genetic heterogeneity [61]. Because the bulk RNA-sequencing results within our data only capture cellular changes at one hour, not 24 h, future research is needed to determine the longer-term transcriptomic effects of (2 *R*,6 *R*)-HNK and reelin. Additionally, our sample size of exclusively female participants limits conclusions that can be drawn to TRD participants more broadly. Previous studies using iPSC-derived neurons nevertheless support the feasibility of this approach [6], revealing important distinctions in response patterns to conventional therapies. For instance, previous iPSC studies found that non-responders to selective serotonin reuptake inhibitors (SSRIs) showed upregulated excitatory serotonergic receptor activity, serotonin-induced hyperactivity, and altered neurite growth and morphology, patterns notably different from those observed in healthy volunteers and treatment responders [8, 9]. These findings suggest that iPSC models can capture clinically relevant differences in treatment response. Healthy control cell lines, as well as cell lines from responders and non-responders to ketamine, should be included in future research studies to determine molecular signalers involved in clinical response to rapid-acting antidepressants, as our small sample size was underpowered to conduct these analyses. Given that individuals with TRD show particularly low response rates to SSRIs, it is possible that molecular alterations induced by ketamine and other rapid-acting agents may be even more readily detectable in iPSC models, in concert with superior clinical outcomes. This enhanced signal-to-noise ratio could make iPSC-derived neurons even more valuable for screening novel rapid-acting antidepressants and understanding their mechanisms of action.

Despite these intriguing preliminary findings, the field of iPSC-derived neuronal modeling for depression is still in its infancy [62]. Our results underscore the potential of this method for therapeutic screening disease modeling and translating relevant discoveries into first-in-human drug development, but further optimization is needed. Future research priorities include: 1) replicating key markers identified here to establish optimal molecular readouts; 2) investigating both earlier and long-term timepoints to assess the durability of treatment effects; and 3) comparing patient-derived cell lines with those from healthy volunteers to identify differential responses, similar to previous studies that found bipolar disorder-specific responses to lithium compared to healthy volunteer cells [63]. Despite their preliminary nature, these findings nevertheless advance the characterization of iPSC-derived neurons from TRD participants and demonstrate their value for translational screening of novel rapid-acting therapeutics.

## Supplementary information

Supplemental Material

## Data Availability

Raw data are available through the Sequence Read Archive (SRA) (https://www.ncbi.nlm.nih.gov/sra). The code used to assess bulk RNA-sequencing data is available upon request.

## Code availability

Raw data are available through the Sequence Read Archive (SRA) (https://www.ncbi.nlm.nih.gov/sra). The code used to assess bulk RNA-sequencing data is available upon request.
