# Supplementary material for: Response of iPSC-derived neurons from individuals with treatment-resistant depression to (2 R,6 R)-hydroxynorketamine and reelin: an exploratory study
Source: Transl Psychiatry. 2025 Nov 18;15:524. doi: 10.1038/s41398-025-03724-6 (PMC12705751; doi:10.1038/s41398-025-03724-6)
Supplement: Supplementary file 1 — Supplemental Material [file 41398_2025_3724_MOESM1_ESM.docx]

*Johnston et al – Response of iPSC-Derived Neurons from Individuals with Treatment-Resistant Depression to (2R,6R)-Hydroxynorketamine and Reelin: An Exploratory Study*

**Supplement**

*Immunocytochemistry*

Immunocytochemical (ICC) analyses were performed to assess protein expression in specific cellular regions (soma, neurite, whole cell) and further validate Western blot results. Given ongoing discussions about the optimal characterization of fluorescent markers, a preliminary assessment was conducted using a modified punctal analysis based on the membrane protein clustering protocol described by Romay-Tallon and colleagues [1]. Following fixation (PFA, 4%), cells designated for ICC analysis were permeabilized by incubation in 0.5% Triton X-100 in PBS for five minutes. To minimize background fluorescence, cells were blocked with 5% BSA for one hour, followed by overnight incubation with primary antibodies (Synapsin I, NR2B, BDNF, TrkB, GluA1, and PSD-95). Appropriate fluorochrome-conjugated secondary antibodies were applied for one hour before coverslips were mounted onto slides for fluorescence imaging. Images were captured on a Nikon A1R confocal microscope at 60x magnification. For analysis, five neurons per coverslip were traced manually and analyzed for region-specific puncta (soma, neurites, whole cell) using an Otsu thresholding approach in Fiji to quantify changes in protein expression. Quantitative measures included punctal count (reflecting the number of protein expression hotspots), average punctal area (as an estimate of expression level), and percent punctal area (representing the proportion of the traced cell that was labeled).

*Immunocytochemical analysis paralleled time-dependent changes in protein expression*

All statistical results are presented in Supplemental Table S1. Immunocytochemical data showed significant differences in protein expression across treatment groups in the whole cell, soma, and neurite compartments. Consistent with Western blot findings, the highest concentrations of reelin (50 nM) and (2*R*,6*R*)-HNK produced the most pronounced increases (at one hour) and decreases (at 24 hours) in protein expression. Notably, NR2B puncta counts were elevated at one hour and reduced at 24 hours in neurites treated with the highest concentrations of reelin and (2*R*,6*R*)-HNK (Supplemental Figure S1). TrkB expression followed a similar pattern. However, these findings should be interpreted with caution given uneven cell populations and binary punctal analyses, which may not completely reflect expression levels.

**Supplemental References**

1. Romay-Tallon R, Rivera-Baltanas T, Allen J, Olivares JM, Kalynchuk LE, Caruncho HJ. Comparative study of two protocols for quantitative image-analysis of serotonin transporter clustering in lymphocytes, a putative biomarker of therapeutic efficacy in major depression. *Biomark Res* 2017; **5:** 27.

**
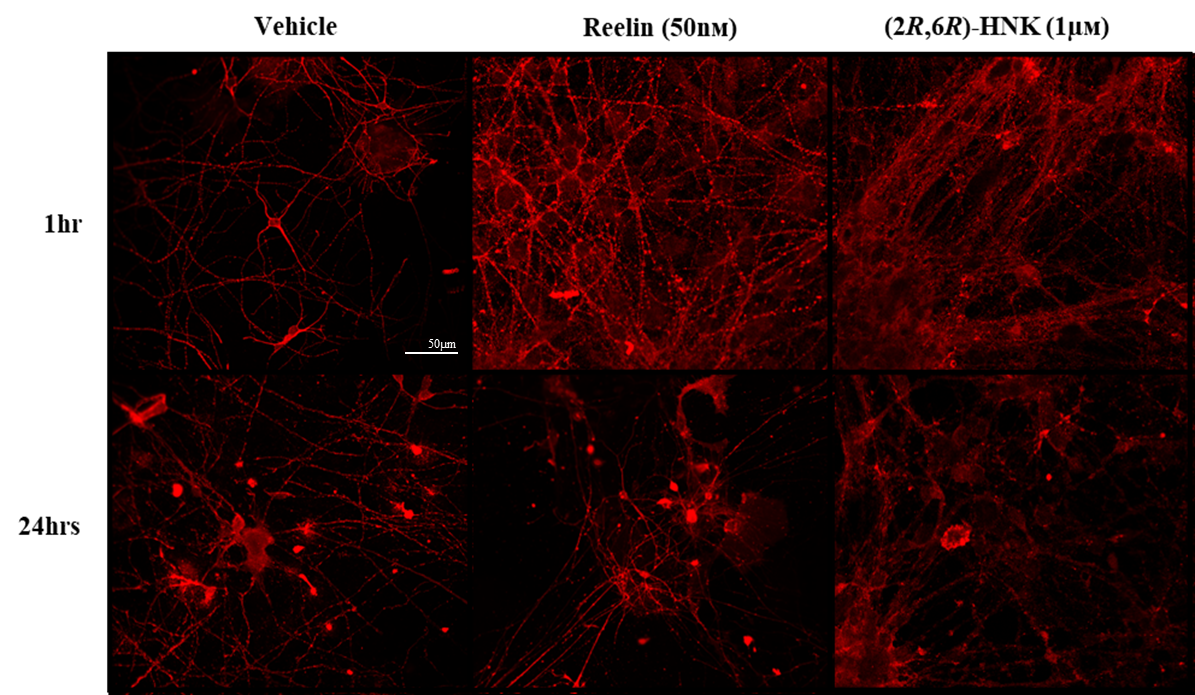
**

**Supplemental Figure S1. Changes in NR2B expression after reelin and (2*R*,6*R*)-hydroxynorketamine (HNK) treatment.** NR2B expression was increased by both the highest concentration of reelin and (2*R*,6*R*)-HNK. Representative images were taken from the same cell line to best reflect drug-induced changes in expression. Images were taken on a Nikon A1R confocal microscope at 60x magnification.

**Supplemental Table S1. Significant differences in cluster size, puncta count, and percent punctal area in immunocytochemical analyses**

| Significance Against Vehicle Control | | | Cluster Size | | | | Puncta Count | | | | % Punctal Area | | | |
| --- | --- | --- | --- | --- | --- | --- | --- | --- | --- | --- | --- | --- | --- | --- |
|  |  |  | Reelin (5nᴍ) | Reelin (10nᴍ) | Reelin (50nᴍ) | HNK (1µᴍ) | Reelin (5nᴍ) | Reelin (10nᴍ) | Reelin (50nᴍ) | HNK (1µᴍ) | Reelin (5nᴍ) | Reelin (10nᴍ) | Reelin (50nᴍ) | HNK (1µᴍ) |
| Whole Cell | BDNF | 1 hour | NS | NS | NS | NS | NS | NS | NS | NS | NS | NS | NS | NS |
|  |  | 24 hour | NS | NS | NS | NS | NS | 0.029 | NS | 0.016 | NS | NS | NS | NS |
|  | TrkB | 1 hour | NS | NS | NS | NS | NS | NS | < 0.001 | 0.015 | NS | NS | NS | NS |
|  |  | 24 hour | NS | NS | NS | NS | NS | NS | NS | 0.021 | NS | NS | NS | NS |
|  | PSD-95 | 1 hour | NS | 0.009 | NS | NS | NS | NS | NS | 0.002 | NS | 0.001 | NS | NS |
|  |  | 24 hour | NS | NS | NS | NS | NS | NS | NS | NS | NS | NS | NS | NS |
|  | GluA1 | 1 hour | NS | NS | NS | NS | 0.031 | 0.002 | NS | NS | NS | NS | NS | NS |
|  |  | 24 hour | NS | NS | NS | NS | NS | NS | NS | NS | NS | NS | NS | NS |
|  | Synapsin I | 1 hour | NS | 0.024 | NS | 0.027 | NS | NS | NS | NS | NS | NS | NS | NS |
|  |  | 24 hour | NS | NS | NS | NS | NS | NS | NS | NS | NS | NS | NS | NS |
|  | NR2B | 1 hour | NS | NS | <0.001 | NS | NS | NS | 0.002 | NS | 0.038 | NS | <0.001 | NS |
|  |  | 24 hour | NS | NS | NS | NS | NS | NS | 0.008 | NS | NS | NS | <0.001 | NS |
| Neurites | BDNF | 1 hour | NS | NS | NS | NS | NS | NS | 0.002 | NS | NS | NS | NS | NS |
|  |  | 24 hour | NS | NS | NS | NS | NS | NS | NS | 0.028 | NS | NS | NS | NS |
|  | TrkB | 1 hour | NS | NS | NS | NS | NS | NS | <0.001 | NS | NS | NS | NS | NS |
|  |  | 24 hour | NS | NS | NS | 0.044 | NS | NS | NS | 0.006 | NS | NS | NS | NS |
|  | PSD-95 | 1 hour | NS | NS | NS | NS | NS | NS | NS | NS | NS | 0.004 | NS | NS |
|  |  | 24 hour | NS | NS | NS | NS | NS | NS | NS | NS | NS | NS | NS | NS |
|  | GluA1 | 1 hour | NS | NS | NS | NS | NS | NS | NS | NS | NS | NS | NS | NS |
|  |  | 24 hour | NS | NS | NS | NS | NS | NS | NS | NS | NS | NS | NS | NS |
|  | Synapsin I | 1 hour | NS | 0.04 | NS | 0.016 | NS | NS | NS | NS | NS | NS | NS | 0.005 |
|  |  | 24 hour | NS | NS | NS | NS | NS | NS | NS | NS | NS | NS | 0.047 | 0.016 |
|  | NR2B | 1 hour | NS | NS | 0.024 | NS | NS | NS | 0.005 | NS | NS | NS | <0.001 | NS |
|  |  | 24 hour | NS | NS | 0.043 | NS | NS | NS | <0.001 | 0.007 | NS | NS | <0.001 | 0.008 |

**All values presented as p-values. NS = Not significant.**

BDNF: brain-derived neurotrophic factor; Trk-B: tyrosine kinase B; PSD-95: postsynaptic density-95 protein; GluA1: glutamate A1 (GluA1) subunit; NR2B: NMDA-receptor subunit-2B

**Supplemental Table S2: Differentially Expressed Genes (DEGs) from Vehicle Control after Reelin (5nᴍ)**

| **gene_name** | **logFC** | **AveExpr** | **t** | **P.Value** | **adj.P.Val** | **B** | **z.std** |
| --- | --- | --- | --- | --- | --- | --- | --- |
| *LINC00710* | -5.5599705 | -2.5519259 | -7.0727601 | 3.2204E-06 | 3.2204E-06 | -2.594383 | -4.6562351 |
| *OGFR-AS1* | -5.5575079 | -3.3777216 | -5.7396636 | 1.5341E-05 | 1.5341E-05 | -2.3871364 | -4.323734 |
| *DNAJC8P4* | -4.9359049 | -5.5620186 | -6.1153471 | 1.8705E-05 | 1.8705E-05 | -1.8771458 | -4.2798142 |
| *GLMP* | -0.6639608 | 3.2916014 | -5.9326661 | 2.6644E-05 | 2.6644E-05 | -2.3786333 | -4.2004008 |
| *ALPK2* | 0.77782252 | 2.83952697 | 5.03432387 | 0.00014568 | 0.00014568 | -2.5733529 | 3.79831925 |
| *GPR52* | -5.2462418 | -5.323325 | -4.6847583 | 0.0001592 | 0.0001592 | -2.3036406 | -3.7762591 |
| *RPS27P25* | 0.85142929 | 2.0066201 | 4.88761077 | 0.0001897 | 0.0001897 | -2.6917167 | 3.73234731 |
| *BCAS1* | -2.9473744 | -1.0709432 | -4.8047646 | 0.00022103 | 0.00022103 | -3.3498496 | -3.6936875 |
| *RNU6-457P* | 3.34829154 | -5.0870771 | 4.67652891 | 0.00028349 | 0.00028349 | -2.6123947 | 3.62993349 |
| *EIF4A1* | -4.5668544 | -1.1334316 | -4.3872534 | 0.0003131 | 0.0003131 | -3.3786809 | -3.6042094 |
| *HMGB1P14* | -4.9394674 | -4.7762225 | -4.5367053 | 0.0003982 | 0.0003982 | -2.7235461 | -3.5412713 |
| *DANT2* | -4.9937577 | -2.4644685 | -4.3762734 | 0.00048559 | 0.00048559 | -3.1015824 | -3.4885823 |
| *FTH1P10* | 4.29239047 | -4.2945613 | 4.52431164 | 0.00048853 | 0.00048853 | -2.7700499 | 3.48696534 |
| *RPS27P3* | 0.46968016 | 2.9631479 | 4.38766266 | 0.00053683 | 0.00053683 | -2.6632197 | 3.46167139 |
| *ALKBH4* | 0.45067158 | 4.07551646 | 4.35148139 | 0.00056501 | 0.00056501 | -2.7800357 | 3.44787978 |
| *MRPS9* | -0.52678 | 3.41304938 | -4.348538 | 0.0005786 | 0.0005786 | -2.7847706 | -3.4414516 |
| *ACOXL-AS1* | -3.1976065 | -0.459069 | -4.306894 | 0.00058829 | 0.00058829 | -3.4082019 | -3.4369553 |
| *CACNA1F* | -4.3049044 | -5.8390916 | -4.3005717 | 0.00059214 | 0.00059214 | -2.6129876 | -3.4351894 |
| *TRPS1* | -0.4791991 | 4.16637003 | -4.2701626 | 0.000671 | 0.000671 | -2.874587 | -3.4011614 |
| *MIR27B* | -3.1225362 | -5.2522459 | -4.1517785 | 0.00081539 | 0.00081539 | -2.8401574 | -3.3475164 |
| *FALEC* | -5.3897751 | -2.6144353 | -3.9190224 | 0.00091379 | 0.00091379 | -3.2659664 | -3.3158082 |
| *LINC00967* | 2.18646826 | -2.7529792 | 4.05687629 | 0.001029 | 0.001029 | -3.0225372 | 3.28247488 |
| *CPSF4L* | -3.1507977 | -3.9829147 | -4.0486566 | 0.00103082 | 0.00103082 | -3.0453618 | -3.2819759 |
| *LINC02777* | 3.07761862 | -0.8349868 | 3.99530719 | 0.00110853 | 0.00110853 | -3.7314105 | 3.26142668 |
| *GDF10* | -0.4686551 | 2.16780455 | -4.0198326 | 0.00111059 | 0.00111059 | -3.0732471 | -3.2608997 |
| *RPS2P32* | -4.7312326 | -1.6099053 | -3.8300723 | 0.00112016 | 0.00112016 | -3.3129984 | -3.2584664 |
| *LMOD1* | 0.95533101 | 0.98687926 | 3.95796325 | 0.00123876 | 0.00123876 | -3.2911403 | 3.22980257 |
| *CENPA* | 0.62291732 | 3.44108338 | 3.94754485 | 0.00134344 | 0.00134344 | -3.0125439 | 3.20653386 |
| *C6orf15* | 2.66634327 | -5.4004824 | 3.89542796 | 0.00138518 | 0.00138518 | -2.9675596 | 3.19772224 |
| *ECI2-DT* | -3.9900011 | -3.6060251 | -3.7280601 | 0.00141455 | 0.00141455 | -3.2469593 | -3.191666 |
| *HAAO* | 1.25183028 | 0.29748646 | 3.84614254 | 0.00155439 | 0.00155439 | -3.1194151 | 3.16433184 |
| *ZC2HC1B* | 3.87549465 | -6.0376708 | 3.61562741 | 0.00182863 | 0.00182863 | -2.9451184 | 3.11673943 |
| *NUP153-AS1* | -3.5839642 | -1.6942913 | -3.7295715 | 0.00190024 | 0.00190024 | -3.3546493 | -3.1053969 |
| *ORMDL1P1* | -3.6443109 | -5.8573853 | -3.5976357 | 0.00190523 | 0.00190523 | -2.9503897 | -3.1046215 |
| *C16orf92* | -3.8646658 | -5.1052913 | -3.7376933 | 0.00190831 | 0.00190831 | -3.0561638 | -3.1041425 |
| *SLC35D3* | -4.1640308 | -4.1896801 | -3.7557272 | 0.00192517 | 0.00192517 | -3.0788114 | -3.101539 |
| *CACNA2D3* | 0.55267483 | 3.68899774 | 3.72081333 | 0.002036 | 0.002036 | -3.1600346 | 3.08493011 |
| *SCRT2* | -0.4924965 | 4.73326975 | -3.7175792 | 0.00204606 | 0.00204606 | -3.0786717 | -3.0834635 |
| *OMG* | -0.9246693 | 2.81270444 | -3.7153575 | 0.00206304 | 0.00206304 | -3.3402469 | -3.0810038 |
| *LINC00184* | 3.82297339 | -4.8998005 | 3.69867483 | 0.00209897 | 0.00209897 | -3.0917372 | 3.07585942 |
| *TTC39C* | 0.44045938 | 3.87428446 | 3.68792065 | 0.00215242 | 0.00215242 | -3.1651154 | 3.06835316 |
| *LETM1P2* | 3.57586593 | -2.9591753 | 3.69420018 | 0.00216391 | 0.00216391 | -3.3773175 | 3.06676199 |
| *TNNT1* | -0.9896648 | 2.24536507 | -3.7059243 | 0.00224338 | 0.00224338 | -3.0962446 | -3.0559636 |
| *RHCE* | -3.6416652 | -0.8627454 | -3.6376416 | 0.00233542 | 0.00233542 | -3.5489901 | -3.0438883 |
| *CTLA4* | -4.3732613 | -4.8364432 | -3.6397397 | 0.00235186 | 0.00235186 | -3.2322663 | -3.0417771 |
| *OASL* | 2.84963591 | -0.6839127 | 3.60859838 | 0.00245143 | 0.00245143 | -3.7901525 | 3.02927287 |
| *SKA3* | 0.38182006 | 3.04283083 | 3.61922959 | 0.00250478 | 0.00250478 | -3.1405261 | 3.02276317 |
| *SLC7A10* | -4.4373781 | -4.0548479 | -3.6211234 | 0.00251164 | 0.00251164 | -3.2778798 | -3.0219355 |
| *EEF1AKMT2* | 0.35982766 | 4.1854371 | 3.60849687 | 0.00256838 | 0.00256838 | -3.1624363 | 3.01516633 |
| *HDDC2* | 0.32471254 | 6.02849259 | 3.59390127 | 0.00264374 | 0.00264374 | -3.1839371 | 3.00638533 |
| *RTKN2* | 0.34133373 | 3.79277407 | 3.58692718 | 0.00267399 | 0.00267399 | -3.10047 | 3.00292454 |
| *CCDC89* | -4.4029684 | -3.486154 | -3.5743743 | 0.00268637 | 0.00268637 | -3.3229304 | -3.0015184 |
| *GOLGA7* | -1.4776707 | 2.26665143 | -3.5863171 | 0.00270138 | 0.00270138 | -3.6656286 | -2.9998218 |
| *LINC02243* | -4.2003619 | -5.4447357 | -3.4438598 | 0.00270343 | 0.00270343 | -3.0850985 | -2.9995903 |
| *IGBP1* | 0.32420284 | 6.08244044 | 3.58284002 | 0.00270722 | 0.00270722 | -3.1724758 | 2.99916391 |
| *LGALS2* | -2.4571642 | -2.276476 | -3.5686791 | 0.00274678 | 0.00274678 | -3.4959666 | -2.9947412 |
| *KCNIP1* | 0.39631834 | 5.01053467 | 3.57065187 | 0.00275903 | 0.00275903 | -3.2346725 | 2.99338303 |
| *COPB2-DT* | -0.511677 | 3.19847409 | -3.569196 | 0.00277187 | 0.00277187 | -3.2206666 | -2.9919662 |
| *PIPOX* | -0.3914404 | 3.62225529 | -3.559658 | 0.00281887 | 0.00281887 | -3.2525607 | -2.9868296 |
| *SEPSECS* | 0.53009892 | 3.97562656 | 3.54988611 | 0.00289381 | 0.00289381 | -3.2154834 | 2.97879816 |
| *AMMECR1L* | 0.32665687 | 5.24641339 | 3.52051534 | 0.00306544 | 0.00306544 | -3.2237917 | 2.96109807 |
| *CENPN* | 0.46881589 | 3.23262605 | 3.51190655 | 0.00315278 | 0.00315278 | -3.2296995 | 2.95243534 |
| *TRPC6* | -4.082058 | -1.175681 | -3.3721845 | 0.00318047 | 0.00318047 | -3.6588277 | -2.9497346 |
| *CLPB* | 0.53149371 | 4.35959975 | 3.50305215 | 0.00320261 | 0.00320261 | -3.2624577 | 2.94759088 |
| *CDC42P4* | 3.4607651 | -5.6651668 | 3.49822103 | 0.00321322 | 0.00321322 | -3.2205921 | 2.94656799 |
| *SMARCD3* | -0.4764468 | 6.28445811 | -3.483725 | 0.00327834 | 0.00327834 | -3.2598847 | -2.9403575 |
| *RPL13AP22* | 1.09210719 | 0.86800587 | 3.45313515 | 0.00341449 | 0.00341449 | -3.3268025 | 2.92772742 |
| *B3GNT7* | 1.16234458 | 0.96800319 | 3.32700437 | 0.00352272 | 0.00352272 | -3.3335726 | 2.91801134 |
| *EQTN* | 3.5034956 | -4.9226136 | 3.43830695 | 0.00355675 | 0.00355675 | -3.234496 | 2.91501161 |
| *CCDC85A* | -3.985972 | -1.7911785 | -3.4182529 | 0.00368221 | 0.00368221 | -3.6493776 | -2.9041765 |
| *FAM149A* | -0.4211343 | 3.64562611 | -3.4168477 | 0.00378131 | 0.00378131 | -3.336566 | -2.8958518 |
| *ALKBH5* | -0.3844879 | 6.29235111 | -3.4017218 | 0.0038994 | 0.0038994 | -3.308615 | -2.886187 |
| *LINC02526* | -4.2176573 | -1.7765098 | -3.2802997 | 0.00391445 | 0.00391445 | -3.6059942 | -2.884975 |
| *SNRPGP17* | 3.31906451 | -5.1866992 | 3.38925644 | 0.00392909 | 0.00392909 | -3.3074702 | 2.88379905 |
| *KLHDC1* | 0.47297857 | 2.40506612 | 3.40163967 | 0.00394123 | 0.00394123 | -3.1845433 | 2.882828 |
| *PSMC1P1* | 1.9028148 | -1.3986925 | 3.3841631 | 0.00394686 | 0.00394686 | -3.2454357 | 2.88237813 |
| *C16orf46-DT* | -4.1827569 | -2.8849826 | -3.4706025 | 0.00408839 | 0.00408839 | -3.4600388 | -2.87126 |
| *ARSJ* | -0.7052819 | 0.45044326 | -3.3734773 | 0.00411901 | 0.00411901 | -3.3342157 | -2.868901 |
| *SLFN13* | 0.81676852 | 2.23854384 | 3.36147039 | 0.00420304 | 0.00420304 | -3.4233364 | 2.86250675 |
| *ACSM5* | -4.4734645 | -4.0873415 | -3.3567935 | 0.00422526 | 0.00422526 | -3.3300455 | -2.8608358 |
| *ADAM18* | -4.4127917 | -5.1107188 | -3.388164 | 0.0042257 | 0.0042257 | -3.2736752 | -2.8608023 |
| *EXOC6* | -0.425352 | 3.94312417 | -3.3632386 | 0.00425871 | 0.00425871 | -3.3368548 | -2.8583348 |
| *DNAJC13* | 0.3327936 | 5.49583317 | 3.36049348 | 0.00426071 | 0.00426071 | -3.3015891 | 2.85818573 |
| *APOL6* | -0.4193437 | 1.46478044 | -3.3542595 | 0.00431184 | 0.00431184 | -3.3195573 | -2.8543989 |
| *RETSAT* | -0.5322563 | 5.29037605 | -3.3489243 | 0.00435324 | 0.00435324 | -3.3694097 | -2.8513619 |
| *SMG8* | -0.5969482 | 2.56982878 | -3.3360566 | 0.00444501 | 0.00444501 | -3.4431159 | -2.8447226 |
| *FABP6* | -4.220296 | -2.276705 | -3.3346397 | 0.00445437 | 0.00445437 | -3.7674446 | -2.8440524 |
| *SPATA4* | -1.2508177 | 0.32832505 | -3.3327877 | 0.00446341 | 0.00446341 | -3.5392547 | -2.8434063 |
| *TMEM184A* | -3.5241047 | -0.9399972 | -3.2201611 | 0.00448215 | 0.00448215 | -3.8709026 | -2.8420712 |
| *NEUROD1* | 0.49283043 | 2.49926048 | 3.33982975 | 0.00449486 | 0.00449486 | -3.4530811 | 2.84116791 |
| *TBX21* | -3.7648969 | -5.0088519 | -3.3194661 | 0.00464802 | 0.00464802 | -3.3983427 | -2.8304663 |
| *DSPP* | -3.2641601 | -5.354223 | -3.3067992 | 0.00478019 | 0.00478019 | -3.3759274 | -2.8214851 |
| *ARHGEF5* | -4.5799536 | -2.7520306 | -3.296982 | 0.0048072 | 0.0048072 | -3.5015504 | -2.8196773 |
| *SLC41A1* | -0.3388046 | 5.40050409 | -3.2950458 | 0.00486218 | 0.00486218 | -3.3777401 | -2.8160255 |
| *CBX3P2* | -2.3454284 | -0.28158 | -3.1781279 | 0.00492591 | 0.00492591 | -3.6015927 | -2.8118391 |
| *REL* | -2.7573127 | -0.6449523 | -3.1728239 | 0.00498487 | 0.00498487 | -3.571667 | -2.8080099 |
| *ZFP30* | 0.43745128 | 5.04012753 | 3.26877262 | 0.00514846 | 0.00514846 | -3.3619197 | 2.79759509 |
| *RPL10P6* | 0.97764787 | 0.54043221 | 3.25004456 | 0.0051841 | 0.0051841 | -3.4053531 | 2.79536612 |
| *TMED4* | -0.2386669 | 6.45723717 | -3.2627161 | 0.0051981 | 0.0051981 | -3.378664 | -2.7944942 |
| *LINC-PINT* | -0.8681636 | 2.71542258 | -3.2580099 | 0.00535553 | 0.00535553 | -3.4564473 | -2.784833 |
| *DGAT2* | -1.8618236 | 0.09269871 | -3.2530158 | 0.00548476 | 0.00548476 | -3.7219819 | -2.7770923 |
| *ENTPD1* | -0.4241341 | 3.54164865 | -3.2358313 | 0.0055465 | 0.0055465 | -3.4295242 | -2.7734523 |
| *SLC7A8* | -0.4187037 | 3.79835881 | -3.2318144 | 0.00555059 | 0.00555059 | -3.4905876 | -2.7732123 |
| *MRPL46* | -1.0752889 | 0.92746502 | -3.2219424 | 0.00558568 | 0.00558568 | -3.4767525 | -2.771161 |
| *GTF2E1* | -0.3333289 | 3.15593477 | -3.2213772 | 0.00563221 | 0.00563221 | -3.4863386 | -2.7684586 |
| *FKBP5* | 0.82143414 | 1.89683282 | 3.11548436 | 0.00566776 | 0.00566776 | -3.4443388 | 2.76640762 |
| *GAPDHP76* | -3.1828456 | -6.0733537 | -3.2045928 | 0.00570898 | 0.00570898 | -3.3499461 | -2.7640443 |
| *OR1F1* | 1.09128214 | 0.07788465 | 3.19961016 | 0.00576973 | 0.00576973 | -3.3950372 | 2.76058845 |
| *ADAL* | 0.41427469 | 3.8967549 | 3.21561485 | 0.00587143 | 0.00587143 | -3.4484789 | 2.75487638 |
| *RPL10P19* | 0.84345519 | 0.79851556 | 3.19884617 | 0.00590151 | 0.00590151 | -3.6667326 | 2.75320389 |
| *CYYR1* | 0.46827479 | 3.84409356 | 3.1903156 | 0.00605409 | 0.00605409 | -3.4362371 | 2.7448371 |
| *COX20P2* | -3.7862922 | -5.5904594 | -3.1592901 | 0.00622369 | 0.00622369 | -3.3760818 | -2.7357572 |
| *FRA10AC1* | 0.34049329 | 4.71595679 | 3.17360233 | 0.00627519 | 0.00627519 | -3.4247306 | 2.73304422 |
| *EVA1A* | 0.86259465 | 0.68565084 | 3.16559961 | 0.00631838 | 0.00631838 | -3.4833552 | 2.73078428 |
| *PRDX2P1* | 3.61528048 | -5.6632837 | 3.15028647 | 0.00632014 | 0.00632014 | -3.3024454 | 2.73069238 |
| *ANP32BP1* | 3.3436151 | -5.3334691 | 3.16570753 | 0.00639905 | 0.00639905 | -3.3949299 | 2.72660052 |
| *ZNF43* | 0.31361443 | 5.59349473 | 3.16172417 | 0.00640644 | 0.00640644 | -3.3958554 | 2.72621927 |
| *TLK2P2* | -3.6319098 | -4.9201442 | -3.1894757 | 0.00647818 | 0.00647818 | -3.4192845 | -2.7225422 |
| *ZNF707* | -0.3756061 | 3.84575058 | -3.1501169 | 0.00657156 | 0.00657156 | -3.4552811 | -2.7178098 |
| *VASH1-AS1* | 3.02267849 | -3.9470986 | 3.14815926 | 0.00659569 | 0.00659569 | -3.4579886 | 2.71659664 |
| *MED22* | -0.2531924 | 5.7205428 | -3.0462812 | 0.0066137 | 0.0066137 | -3.422735 | -2.7156941 |
| *RDH10-AS1* | -3.6453411 | -4.9415593 | -3.131986 | 0.00661831 | 0.00661831 | -3.3962571 | -2.7154631 |
| *RPL10P16* | 0.56747585 | 2.31154248 | 3.12117614 | 0.00671488 | 0.00671488 | -3.3909167 | 2.71066324 |
| *PLK4* | 0.55173229 | 3.37530736 | 3.13855389 | 0.00673237 | 0.00673237 | -3.4431266 | 2.70980027 |
| *ZNF823* | 0.43239646 | 2.69837115 | 3.13558431 | 0.00677726 | 0.00677726 | -3.4013258 | 2.70759547 |
| *SLFN12* | 0.80624315 | 1.54961421 | 3.13138014 | 0.00683745 | 0.00683745 | -3.3577608 | 2.70465911 |
| *PDE7B-AS1* | -4.3169251 | -3.6570102 | -3.0313201 | 0.00683748 | 0.00683748 | -3.4249417 | -2.7046577 |
| *AKR1B10* | 4.05930115 | -5.7891894 | 3.03025982 | 0.00685362 | 0.00685362 | -3.3666977 | 2.70387453 |
| *RORB* | 0.4827369 | 2.79251306 | 3.12610969 | 0.00691437 | 0.00691437 | -3.473884 | 2.70094045 |
| *CNKSR3* | -0.4766669 | 2.91859795 | -3.1334536 | 0.00694147 | 0.00694147 | -3.4240861 | -2.6996394 |
| *OR7E116P* | 3.85429599 | -5.0133808 | 3.12458203 | 0.00696016 | 0.00696016 | -3.4141032 | 2.69874432 |
| *IFI35* | 0.4755648 | 3.01399562 | 3.11298674 | 0.00706558 | 0.00706558 | -3.4706095 | 2.69373749 |
| *RPS23P8* | 0.60922342 | 1.67616528 | 3.10458883 | 0.00712198 | 0.00712198 | -3.4517655 | 2.69108606 |
| *NLRP14* | -4.0757683 | -4.252015 | -3.1152829 | 0.00713791 | 0.00713791 | -3.4419169 | -2.6903407 |
| *IL21R* | -4.9062238 | -2.6817638 | -3.0972545 | 0.007172 | 0.007172 | -3.6588521 | -2.6887503 |
| *TRMT10B* | 0.31197455 | 3.54783005 | 3.10689687 | 0.0071762 | 0.0071762 | -3.4222751 | 2.68855528 |
| *RPSAP52* | -3.4526959 | -5.2863891 | -3.0838286 | 0.00722455 | 0.00722455 | -3.4548069 | -2.6863125 |
| *FIBIN* | -0.4043263 | 2.84432528 | -3.1019642 | 0.0072592 | 0.0072592 | -3.5206565 | -2.6847136 |
| *UNC50* | -0.3672326 | 4.59411408 | -3.0983875 | 0.00728904 | 0.00728904 | -3.5091688 | -2.6833421 |
| *GPR183* | -3.5372945 | -4.0811579 | -3.0828408 | 0.00738392 | 0.00738392 | -3.5716882 | -2.6790146 |
| *ST3GAL6* | 0.64092817 | 1.8735818 | 3.09731963 | 0.0074359 | 0.0074359 | -3.5916218 | 2.67666465 |
| *AVPR2* | -2.8963056 | -4.8760106 | -3.0903483 | 0.00744512 | 0.00744512 | -3.5281327 | -2.6762495 |
| *QPCT* | -0.5228499 | 2.92308896 | -3.0872433 | 0.00744655 | 0.00744655 | -3.5498084 | -2.6761853 |
| *HMGN5* | -0.3009843 | 3.97980641 | -3.0803949 | 0.007512 | 0.007512 | -3.4912977 | -2.673251 |
| *TUSC1* | -0.3323668 | 4.54373094 | -3.0874998 | 0.00752724 | 0.00752724 | -3.4894618 | -2.6725711 |
| *ASB14* | 1.18020184 | -0.5521644 | 3.12335543 | 0.00754737 | 0.00754737 | -3.3444774 | 2.67167515 |
| *MIR4482* | -3.4294969 | -4.9415274 | -2.9847463 | 0.00758206 | 0.00758206 | -3.4223061 | -2.6701356 |
| *CHKA-DT* | -1.4741181 | -0.1677426 | -3.0690259 | 0.00760324 | 0.00760324 | -3.6587498 | -2.6691989 |
| *HCG14* | -4.36962 | -5.1477938 | -2.9805447 | 0.00765296 | 0.00765296 | -3.4447745 | -2.6670089 |
| *MBD2* | 0.319564 | 5.05724567 | 3.07493767 | 0.00767014 | 0.00767014 | -3.4773779 | 2.66625548 |
| *ICAM5* | -0.7456612 | 3.53375423 | -3.068977 | 0.00769021 | 0.00769021 | -3.5426494 | -2.6653766 |
| *CNIH4* | 0.20133891 | 5.25410771 | 3.06445189 | 0.00781286 | 0.00781286 | -3.4797727 | 2.66005185 |
| *COQ8A* | -4.2355964 | -3.1394578 | -3.0832532 | 0.007866 | 0.007866 | -3.6835672 | -2.6577681 |
| *RPL17P38* | -3.4746337 | -3.9974894 | -3.054167 | 0.00797325 | 0.00797325 | -3.6192331 | -2.6532003 |
| *SMAD9* | 0.5958835 | 4.04156991 | 3.05014598 | 0.0080173 | 0.0080173 | -3.4955815 | 2.65134053 |
| *MIR1244-3* | 1.95370635 | -1.3936698 | 3.0337589 | 0.00810947 | 0.00810947 | -3.5950315 | 2.64747783 |
| *SEZ6* | -0.2546886 | 6.36735629 | -3.0407841 | 0.00820727 | 0.00820727 | -3.4996653 | -2.6434221 |
| *UTP14A* | 0.446357 | 3.51082802 | 3.03795093 | 0.00826909 | 0.00826909 | -3.4959138 | 2.64088051 |
| *CDKL5* | 0.46944186 | 3.84364132 | 3.04380947 | 0.00828714 | 0.00828714 | -3.535412 | 2.64014145 |
| *LMNTD1* | -1.410556 | -5.8616685 | -3.0321632 | 0.00830726 | 0.00830726 | -3.4968427 | -2.6393199 |
| *SURF4* | -0.3122711 | 7.30196085 | -3.0323621 | 0.00834821 | 0.00834821 | -3.5194769 | -2.6376522 |
| *LBX2-AS1* | -4.5941407 | -2.7587835 | -3.037497 | 0.00838908 | 0.00838908 | -3.7122103 | -2.6359957 |
| *ITFG2-AS1* | 2.75524178 | -5.5829525 | 3.02188755 | 0.00846658 | 0.00846658 | -3.4774399 | 2.63287396 |
| *LINC01585* | -3.2565393 | -5.5840476 | -3.1085095 | 0.008514 | 0.008514 | -3.372406 | -2.6309763 |
| *PRH1* | 3.23208739 | -1.7976461 | 2.93118196 | 0.00853544 | 0.00853544 | -3.5584883 | 2.63012117 |
| *PRADC1P1* | -3.923191 | -4.0478113 | -2.929288 | 0.00857119 | 0.00857119 | -3.6409734 | -2.6287002 |
| *CTSS* | 0.79152815 | 1.82523343 | 3.06081808 | 0.0085932 | 0.0085932 | -3.4632962 | 2.6278278 |
| *RNF216-IT1* | -3.6090136 | -4.4063833 | -3.0165226 | 0.00861406 | 0.00861406 | -3.5237435 | -2.6270031 |
| *GDPD2* | -0.4334785 | 5.82918643 | -3.015818 | 0.00863045 | 0.00863045 | -3.5466247 | -2.6263561 |
| *TAF5L* | -0.3768316 | 4.47047136 | -3.01564 | 0.00865582 | 0.00865582 | -3.4902238 | -2.6253567 |
| *FOXC1* | -0.9323759 | 1.24724926 | -3.0305415 | 0.00869481 | 0.00869481 | -3.6480932 | -2.6238263 |
| *IDNK* | 0.38475549 | 4.41722596 | 3.01062618 | 0.00870812 | 0.00870812 | -3.5071398 | 2.62330518 |
| *TUBB3* | -0.6068032 | 3.31543774 | -3.0063246 | 0.00885007 | 0.00885007 | -3.537443 | -2.6177923 |
| *MXD1* | -0.5956746 | 3.51958396 | -2.9979515 | 0.00893826 | 0.00893826 | -3.6458907 | -2.6144064 |
| *LINC02362* | 4.5405999 | -4.8999709 | 3.00266136 | 0.00896263 | 0.00896263 | -3.4506851 | 2.61347642 |
| *SGCG* | -0.7157677 | -0.0486781 | -2.9935158 | 0.00907474 | 0.00907474 | -3.6098071 | -2.6092256 |
| *ZNF18* | 0.50203758 | 4.00780791 | 2.99005382 | 0.0091219 | 0.0091219 | -3.5233537 | 2.60745157 |
| *HAR1B* | -3.9809259 | -2.8260371 | -2.9968323 | 0.00920037 | 0.00920037 | -3.5735145 | -2.6045174 |
| *PIGBOS1* | 0.66487998 | 3.06032534 | 2.97340689 | 0.00921787 | 0.00921787 | -3.5979388 | 2.60386631 |
| *ZIC4* | 0.39956115 | 3.69555649 | 2.98123498 | 0.00925186 | 0.00925186 | -3.5544752 | 2.60260443 |
| *BTBD16* | -3.9361073 | -3.4238265 | -2.8858765 | 0.00943116 | 0.00943116 | -3.640494 | -2.5960164 |
| *SGSM3* | -1.0606515 | -0.414203 | -2.9803662 | 0.00943684 | 0.00943684 | -3.6170332 | -2.5958094 |
| *LRRC9* | -0.4689018 | 2.69262783 | -2.9693713 | 0.00946113 | 0.00946113 | -3.5568566 | -2.5949258 |
| *ANGPT4* | -3.8435681 | -3.7271993 | -2.9732261 | 0.00954703 | 0.00954703 | -3.6752806 | -2.591818 |
| *CLSPN* | -0.7709787 | 2.28317757 | -2.9769634 | 0.00957183 | 0.00957183 | -3.5444148 | -2.5909253 |
| *CSP2* | 3.63622683 | -3.4978593 | 2.9406763 | 0.00967654 | 0.00967654 | -3.5558151 | 2.58717862 |
| *NACA4P* | 3.01088645 | -3.9231729 | 2.87219385 | 0.00971901 | 0.00971901 | -3.6240937 | 2.58566958 |
| *ADAMTSL4-AS2* | -3.0163804 | -1.5542433 | -2.9430178 | 0.00972718 | 0.00972718 | -3.6930009 | -2.5853798 |
| *KDELR1* | -0.2980898 | 7.84632283 | -2.9570372 | 0.00973253 | 0.00973253 | -3.5566237 | -2.5851902 |
| *KRT8P33* | -4.3729173 | -3.2114966 | -2.9635715 | 0.00975913 | 0.00975913 | -3.5721406 | -2.5842489 |
| *SLC2A14* | 2.40069396 | -2.5949959 | 2.95571017 | 0.00978044 | 0.00978044 | -3.5882545 | 2.58349688 |
| *VSX2* | 1.89003254 | -3.5852545 | 2.94756594 | 0.00990065 | 0.00990065 | -3.5058275 | 2.57928006 |
| *MRPL13* | -0.3565204 | 4.88731158 | -2.9470071 | 0.0099214 | 0.0099214 | -3.5613063 | -2.5785567 |
| *PCDHA12* | -3.0327072 | -0.0895018 | -2.8622014 | 0.00993454 | 0.00993454 | -3.9603264 | -2.5780995 |

**Supplemental Table S3: Differentially Expressed Genes (DEGs) from Vehicle Control after Reelin (10 nᴍ)**

| **gene_name** | **logFC** | **AveExpr** | **t** | **P.Value** | **adj.P.Val** | **B** | **z.std** |
| --- | --- | --- | --- | --- | --- | --- | --- |
| *TSPAN14-AS1* | 4.13782137 | -5.0135153 | 6.04400767 | 2.0836E-05 | 2.0836E-05 | -3.038689 | 4.25573619 |
| *DNAJC8P4* | -5.0440101 | -5.5620186 | -5.7364525 | 3.379E-05 | 3.379E-05 | -3.1768998 | -4.1462967 |
| *SKOR2* | 0.7899792 | -3.863132 | 5.53059146 | 5.6156E-05 | 5.6156E-05 | -3.1841188 | 4.02840589 |
| *PLA2G12A* | 0.46896971 | 5.01458822 | 5.04560812 | 0.00013533 | 0.00013533 | -3.3769995 | 3.81655856 |
| *ZNF613* | 0.614979 | 2.82874958 | 4.97340021 | 0.00016394 | 0.00016394 | -3.3920649 | 3.76893996 |
| *NAPA-AS1* | -2.0157466 | -0.3223807 | -4.932176 | 0.00018166 | 0.00018166 | -3.598227 | -3.7432446 |
| *GAPDHP63* | 1.4565559 | -0.2199493 | 4.59945083 | 0.00030061 | 0.00030061 | -3.353349 | 3.61477555 |
| *APOL6* | -0.5927437 | 1.46478044 | -4.5860976 | 0.0003506 | 0.0003506 | -3.4430598 | -3.5747232 |
| *ENPP7P12* | 5.72972234 | -4.6441548 | 4.2536523 | 0.00042481 | 0.00042481 | -3.3831891 | 3.52417142 |
| *TNNT3* | -6.4882355 | -5.3550568 | -4.3505262 | 0.00052726 | 0.00052726 | -3.6151184 | -3.4665127 |
| *CFAP97D1* | 4.14072108 | -3.1562764 | 4.24122879 | 0.00063098 | 0.00063098 | -3.6565763 | 3.41793712 |
| *PPIAL4A* | 4.78398693 | -5.1517854 | 4.01745752 | 0.00072937 | 0.00072937 | -3.4378962 | 3.37829561 |
| *GLMP* | -0.458517 | 3.2916014 | -4.2176339 | 0.0007343 | 0.0007343 | -3.5502056 | -3.3764422 |
| *ARAP1-AS2* | 4.79911298 | -5.4765194 | 4.09122813 | 0.00086582 | 0.00086582 | -3.502683 | 3.33084914 |
| *TMEM80* | -0.3227315 | 4.73962785 | -4.0986131 | 0.00093492 | 0.00093492 | -3.6494071 | -3.3094134 |
| *CHRND* | -6.3775863 | -4.7568679 | -4.1131957 | 0.00095612 | 0.00095612 | -3.6981021 | -3.3031311 |
| *FOSL2-AS1* | -3.824157 | -4.5000861 | -4.0643161 | 0.00098992 | 0.00098992 | -3.7409409 | -3.2933749 |
| *TLK2P2* | -5.1795078 | -4.9201442 | -4.1091616 | 0.0010258 | 0.0010258 | -3.6822022 | -3.2833546 |
| *RPL23AP81* | -3.4526462 | -4.2469773 | -3.966715 | 0.00120231 | 0.00120231 | -3.7308239 | -3.2383307 |
| *GLYATL2* | -1.3633013 | 0.46493764 | -3.9912844 | 0.00121417 | 0.00121417 | -3.9551108 | -3.2355301 |
| *ALOXE3P1* | -3.8172044 | -5.925008 | -3.9379452 | 0.00129237 | 0.00129237 | -3.6424496 | -3.2176698 |
| *LINC01585* | -4.6812331 | -5.5840476 | -4.0649809 | 0.00132309 | 0.00132309 | -3.5782071 | -3.2109247 |
| *AUNIP* | -1.1914784 | 1.44822527 | -3.7209965 | 0.00143757 | 0.00143757 | -3.8806323 | -3.1869991 |
| *LINC02447* | 2.61612617 | -1.5829321 | 3.86693761 | 0.00146241 | 0.00146241 | -3.7296254 | 3.18204159 |
| *PSPHP1* | -0.4228629 | -3.1163877 | -3.8280327 | 0.00162618 | 0.00162618 | -3.6669424 | -3.1511703 |
| *HOXB3* | 3.86675813 | -3.1487424 | 3.8206361 | 0.00167078 | 0.00167078 | -3.9022232 | 3.14325957 |
| *MCMDC2* | 0.71022896 | 0.84060523 | 3.73875708 | 0.00193502 | 0.00193502 | -3.5669346 | 3.10002909 |
| *MIR2115* | 3.83013866 | -5.8403014 | 3.6987037 | 0.00195359 | 0.00195359 | -3.6339885 | 3.0971987 |
| *HID1-AS1* | 4.29204844 | -5.9594707 | 3.68067028 | 0.0019832 | 0.0019832 | -3.5900898 | 3.09273649 |
| *LINC02765* | -5.1701881 | -4.2371853 | -3.7101239 | 0.0020099 | 0.0020099 | -3.8309417 | -3.0887662 |
| *RDH10-AS1* | -4.6027628 | -4.9415593 | -3.6325575 | 0.0022364 | 0.0022364 | -3.7382357 | -3.056898 |
| *SCUBE2* | 0.48291728 | 3.9590801 | 3.66449071 | 0.00226276 | 0.00226276 | -3.7354023 | 3.05338487 |
| *C1QTNF1* | -2.0848536 | -0.8453654 | -3.6453126 | 0.00227958 | 0.00227958 | -3.9510413 | -3.0511609 |
| *SNRPFP1* | 3.67416284 | -4.3066104 | 3.62641904 | 0.00238482 | 0.00238482 | -3.7085734 | 3.03758465 |
| *TLX1* | 1.75877598 | -4.5111866 | 3.62432514 | 0.00245826 | 0.00245826 | -3.7281131 | 3.02843212 |
| *RPL10P19* | 0.96069484 | 0.79851556 | 3.62255267 | 0.0024587 | 0.0024587 | -3.9161293 | 3.02837803 |
| *SNRPGP10* | -0.7589293 | 1.68194253 | -3.5993138 | 0.00253098 | 0.00253098 | -3.8562744 | -3.019613 |
| *SLC35D3* | -4.5712198 | -4.1896801 | -3.5814735 | 0.00267807 | 0.00267807 | -3.8620509 | -3.00246 |
| *EVA1A* | 0.91355867 | 0.68565084 | 3.55243859 | 0.00284309 | 0.00284309 | -3.6711756 | 2.98421244 |
| *NUDCP2* | 3.75146462 | -3.5302708 | 3.42037517 | 0.00285141 | 0.00285141 | -3.7949959 | 2.98331835 |
| *GPR52* | -4.0981863 | -5.323325 | -3.4185735 | 0.00286309 | 0.00286309 | -3.7223958 | -2.9820675 |
| *NAALADL1* | -5.2166759 | -3.6227846 | -3.514867 | 0.00290955 | 0.00290955 | -3.9453574 | -2.9771355 |
| *C16orf92* | -3.9577144 | -5.1052913 | -3.5195255 | 0.00294129 | 0.00294129 | -3.7905421 | -2.9738076 |
| *CXCL10* | -0.7890916 | -3.0424001 | -3.5307258 | 0.0029924 | 0.0029924 | -3.8871147 | -2.9685176 |
| *PTMAP4* | 2.46488581 | -2.461143 | 3.39465945 | 0.0030226 | 0.0030226 | -3.7854214 | 2.96542969 |
| *VPS9D1-AS1* | -1.5951467 | 0.36276202 | -3.4830828 | 0.0030862 | 0.0030862 | -3.9693647 | -2.9590193 |
| *RPSAP54* | 1.80906384 | -1.4715367 | 3.49253108 | 0.00317808 | 0.00317808 | -3.8891478 | 2.94996652 |
| *H19* | -0.897452 | -0.9213935 | -3.499171 | 0.00320501 | 0.00320501 | -3.8525483 | -2.9473589 |
| *MCEE* | 0.65186456 | 3.14858151 | 3.47994494 | 0.00332482 | 0.00332482 | -3.7805979 | 2.93599241 |
| *FAM87A* | -4.6158457 | -4.3096442 | -3.454191 | 0.00334254 | 0.00334254 | -3.9021738 | -2.9343439 |
| *ZNF568* | 0.42113605 | 3.77984358 | 3.47546619 | 0.00335968 | 0.00335968 | -3.8421292 | 2.93275594 |
| *HOXB9* | 1.13486411 | -5.7953785 | 3.47010343 | 0.00338118 | 0.00338118 | -3.785732 | 2.93077436 |
| *MIR27B* | -2.7522922 | -5.2522459 | -3.4579627 | 0.00341255 | 0.00341255 | -3.779162 | -2.9279046 |
| *GBGT1* | 4.83217264 | -2.6759364 | 3.46538127 | 0.00341994 | 0.00341994 | -3.8970206 | 2.92723152 |
| *CD163* | -4.0889675 | -3.7292928 | -3.4558079 | 0.0034298 | 0.0034298 | -3.9666874 | -2.9263364 |
| *GCOM1* | -1.0187667 | -5.7262293 | -3.4626715 | 0.00343847 | 0.00343847 | -3.8024493 | -2.925551 |
| *SLFN13* | 0.86359035 | 2.23854384 | 3.44649382 | 0.00350985 | 0.00350985 | -3.8601077 | 2.91915211 |
| *KCNK18* | -4.2846906 | -5.3891239 | -3.4028406 | 0.00378739 | 0.00378739 | -3.8793541 | -2.8953473 |
| *LINC01353* | -5.3270703 | -4.061985 | -3.4012856 | 0.00386897 | 0.00386897 | -3.9298533 | -2.8886519 |
| *ATP6V0C* | 3.13977586 | -2.3242885 | 3.40306371 | 0.00387093 | 0.00387093 | -3.9116178 | 2.88849276 |
| *SETP20* | -4.3027023 | -5.1345263 | -3.3919093 | 0.00393176 | 0.00393176 | -3.8167311 | -2.8835855 |
| *OR5K2* | -3.2774815 | -4.9128634 | -3.3959186 | 0.0039341 | 0.0039341 | -3.925369 | -2.8833977 |
| *CDC37L1-DT* | 1.79167257 | 0.00127962 | 3.36321359 | 0.00402965 | 0.00402965 | -3.8807107 | 2.87583128 |
| *LINC02777* | 2.67387958 | -0.8349868 | 3.35536207 | 0.00417033 | 0.00417033 | -4.2133157 | 2.86498217 |
| *ZNF154* | -0.3584889 | 3.69358923 | -3.3509589 | 0.00432863 | 0.00432863 | -3.8586269 | -2.8531637 |
| *ACTBP7* | 4.75752776 | -3.6889813 | 3.22620742 | 0.00442161 | 0.00442161 | -3.7880976 | 2.84640338 |
| *CTRB1* | 3.75748612 | -3.9666831 | 3.29412515 | 0.00448918 | 0.00448918 | -3.8201699 | 2.84157159 |
| *KLC2-AS1* | 2.90854173 | -5.0209242 | 3.3128078 | 0.00450439 | 0.00450439 | -3.7231201 | 2.84049304 |
| *VN1R2* | 2.9087389 | -5.4672605 | 3.31815341 | 0.00460972 | 0.00460972 | -3.8667268 | 2.83311219 |
| *RPL21P37* | 3.82589139 | -5.3543104 | 3.29363708 | 0.00461134 | 0.00461134 | -3.8166456 | 2.83299958 |
| *PTMAP5* | -1.0183565 | 0.84668124 | -3.2959005 | 0.00473857 | 0.00473857 | -3.9698259 | -2.8242889 |
| *ZNF678* | -0.4191977 | 3.93118773 | -3.3007177 | 0.00480761 | 0.00480761 | -3.9043721 | -2.81965 |
| *MYPN* | -4.086593 | -5.8463109 | -3.3039318 | 0.00482602 | 0.00482602 | -3.8309028 | -2.818423 |
| *HGD* | -1.4515316 | 0.60809311 | -3.3023785 | 0.00487973 | 0.00487973 | -4.0452228 | -2.8148679 |
| *FGF14* | 0.45825965 | 3.43592608 | 3.28052446 | 0.00503623 | 0.00503623 | -3.8583465 | 2.80470745 |
| *LRTM1* | -3.2856186 | -4.3639857 | -3.2622622 | 0.005125 | 0.005125 | -3.8900699 | -2.7990703 |
| *ZNF474* | -1.1429716 | 1.2611895 | -3.2571014 | 0.00520114 | 0.00520114 | -4.047304 | -2.7943048 |
| *PSMD14-DT* | -4.6892109 | -5.2932543 | -3.3207387 | 0.00530746 | 0.00530746 | -3.8305011 | -2.7877554 |
| *AHI1-DT* | -0.7339301 | 1.4734831 | -3.2422353 | 0.0054199 | 0.0054199 | -4.002391 | -2.7809564 |
| *TVP23A* | -0.5832459 | 3.28753904 | -3.2395194 | 0.00544672 | 0.00544672 | -3.9622159 | -2.7793536 |
| *PRAME* | 0.91362072 | -4.9291064 | 3.23356588 | 0.0054935 | 0.0054935 | -3.7770998 | 2.77657481 |
| *NCMAP* | -4.6594778 | -4.8686188 | -3.1964667 | 0.00558547 | 0.00558547 | -3.8966172 | -2.7711733 |
| *SH3GL1P1* | 0.7780266 | 1.71540548 | 3.22647208 | 0.00562094 | 0.00562094 | -3.8622873 | 2.76911134 |
| *LMOD1* | 0.78072131 | 0.98687926 | 3.22101014 | 0.00564421 | 0.00564421 | -3.9882721 | 2.76776512 |
| *LGALS2* | -2.3945293 | -2.276476 | -3.2183861 | 0.00564613 | 0.00564613 | -4.0791943 | -2.7676544 |
| *KCNIP1-AS1* | 3.99162925 | -5.1760543 | 3.19978145 | 0.00568523 | 0.00568523 | -3.9238244 | 2.76540424 |
| *CLEC18A* | 0.68704918 | 1.25255885 | 3.20806629 | 0.00579154 | 0.00579154 | -3.8792549 | 2.75935621 |
| *RPS27P9* | 2.96975666 | -4.2602025 | 3.19822365 | 0.00579609 | 0.00579609 | -3.8395212 | 2.75909953 |
| *BALR6* | -4.3647114 | -4.7852743 | -3.1031797 | 0.00582575 | 0.00582575 | -3.8976342 | -2.7574309 |
| *LINC01965* | -2.4515597 | -3.0267855 | -3.1727349 | 0.00627459 | 0.00627459 | -4.141789 | -2.7330756 |
| *RTN4RL1* | 0.72168905 | 2.06708486 | 3.18129462 | 0.00635529 | 0.00635529 | -3.8244566 | 2.72886388 |
| *NMRK2* | -4.4268759 | -3.0716893 | -3.212228 | 0.00637043 | 0.00637043 | -4.0690418 | -2.7280794 |
| *RPS28P7* | 0.83441229 | 1.05237821 | 3.16442574 | 0.00638406 | 0.00638406 | -3.870937 | 2.72737414 |
| *TRIM63* | -4.4161484 | -4.8819229 | -3.1277622 | 0.00646884 | 0.00646884 | -3.9272614 | -2.7230187 |
| *UBXN7-AS1* | 3.8489163 | -3.9021307 | 3.03587465 | 0.00676859 | 0.00676859 | -3.9346368 | 2.70802018 |
| *ZNF662* | -3.5178569 | -4.7969996 | -3.1274105 | 0.00678829 | 0.00678829 | -3.9383375 | -2.7070556 |
| *MAP3K1* | -0.5963168 | 2.76560075 | -3.1304851 | 0.00689622 | 0.00689622 | -4.043111 | -2.7018147 |
| *VASH1-DT* | -3.7393903 | -5.4845698 | -3.1105358 | 0.00689994 | 0.00689994 | -3.9391746 | -2.7016354 |
| *LINC01485* | -4.1741112 | -5.3622505 | -3.1286734 | 0.00694363 | 0.00694363 | -3.9146799 | -2.6995359 |
| *TMEM185AP1* | 3.96590331 | -5.2345258 | 3.10786269 | 0.00696132 | 0.00696132 | -3.8789158 | 2.69868896 |
| *FAM124B* | -4.4200659 | -3.1372553 | -3.1192298 | 0.00697464 | 0.00697464 | -4.0689871 | -2.6980525 |
| *S1PR1-DT* | -1.0827398 | 0.51583317 | -3.1220424 | 0.00707248 | 0.00707248 | -3.9966251 | -2.6934118 |
| *ZEB2P1* | -3.6732983 | -5.0601433 | -3.0988105 | 0.00712428 | 0.00712428 | -3.9809284 | -2.6909783 |
| *POF1B* | 3.3772779 | -5.0481555 | 3.09132753 | 0.0071314 | 0.0071314 | -3.9222109 | 2.69064503 |
| *SELENBP1* | -0.3433105 | 4.81670517 | -3.1058535 | 0.00716724 | 0.00716724 | -3.9626039 | -2.6889722 |
| *SAMD4A-AS1* | -3.946074 | -3.8145871 | -3.0875304 | 0.00738088 | 0.00738088 | -4.1295937 | -2.6791525 |
| *SNORD83A* | 3.79313776 | -5.0274776 | 3.0506313 | 0.00758353 | 0.00758353 | -3.8841334 | 2.67007044 |
| *SMG8* | -0.5821669 | 2.56982878 | -3.0663103 | 0.0077137 | 0.0077137 | -4.0082055 | -2.6643511 |
| *MYO1B-AS1* | -4.8721464 | -4.6458845 | -3.0359977 | 0.00782926 | 0.00782926 | -3.9317328 | -2.6593456 |
| *RNU6-457P* | 2.40529669 | -5.0870771 | 3.05073402 | 0.00787003 | 0.00787003 | -3.9704552 | 2.65759554 |
| *LNC-LBCS* | -0.9342719 | 2.14626643 | -3.0601853 | 0.00789418 | 0.00789418 | -4.0158962 | -2.6565625 |
| *RPP38* | -0.5344318 | 2.90536758 | -3.0578465 | 0.00789964 | 0.00789964 | -3.9588601 | -2.6563295 |
| *RPS6KA6* | 0.4504227 | 3.91906053 | 3.05234494 | 0.00803994 | 0.00803994 | -3.9598602 | 2.65038806 |
| *KLF8* | 0.57214663 | 2.68536117 | 3.03884524 | 0.0081596 | 0.0081596 | -3.9471073 | 2.64539336 |
| *SNORA31* | -4.0253219 | -4.567629 | -2.9402558 | 0.00836618 | 0.00836618 | -3.9533465 | -2.6369231 |
| *LINC02055* | -4.3408068 | -4.8343786 | -3.0071835 | 0.0084867 | 0.0084867 | -3.9921845 | -2.6320673 |
| *FAM66D* | 0.53338692 | 3.48720002 | 3.0219888 | 0.00849608 | 0.00849608 | -3.9433059 | 2.63169238 |
| *MIR122HG* | -3.8707582 | -5.2020767 | -3.0140607 | 0.00855144 | 0.00855144 | -4.0269297 | -2.6294848 |
| *VWC2* | 0.69170946 | 2.27867452 | 3.01638169 | 0.00861076 | 0.00861076 | -3.9119121 | 2.62713334 |
| *TEDC2-AS1* | -4.6282626 | -2.8563425 | -2.925425 | 0.00864454 | 0.00864454 | -4.0787555 | -2.6258007 |
| *LINC02552* | 3.72362822 | -4.3395556 | 3.00663719 | 0.00873343 | 0.00873343 | -3.9530341 | 2.62231636 |
| *KCNIP1* | 0.35141376 | 5.01053467 | 3.00702702 | 0.0087826 | 0.0087826 | -3.9707803 | 2.62040281 |
| *SPP2* | -3.7057843 | -5.4422612 | -2.9975362 | 0.00881759 | 0.00881759 | -3.9816013 | -2.6190464 |
| *TNFAIP6* | 2.11787841 | -3.2750065 | 2.99741072 | 0.00896577 | 0.00896577 | -3.9912673 | 2.61335643 |
| *AKR1E2* | 2.39724236 | -0.2926719 | 2.90469271 | 0.00904867 | 0.00904867 | -4.059346 | 2.61020972 |
| *TMEM11-DT* | -3.511736 | -1.9389096 | -2.900286 | 0.00913689 | 0.00913689 | -4.0459363 | -2.6068894 |
| *CPSF4L* | -2.8189408 | -3.9829147 | -2.9763302 | 0.00922794 | 0.00922794 | -4.1305855 | -2.603492 |
| *ALOX5AP* | 4.22095144 | -5.730754 | 2.89104381 | 0.0093246 | 0.0093246 | -3.872325 | 2.59991829 |
| *GAPDHP76* | -3.1663507 | -6.0733537 | -2.9505364 | 0.00949963 | 0.00949963 | -3.9582717 | -2.5935299 |
| *KRT17P3* | 3.69537423 | -5.7049773 | 2.87786863 | 0.00959861 | 0.00959861 | -3.910969 | 2.5899635 |
| *FLG* | -1.5662003 | -5.6871039 | -2.9597399 | 0.00962129 | 0.00962129 | -3.9794621 | -2.5891511 |
| *PPIAP42* | -3.3907151 | -5.0661646 | -2.8747341 | 0.00966493 | 0.00966493 | -3.9162147 | -2.5875921 |
| *INSYN1-AS1* | 0.9407882 | 1.53349167 | 2.93859962 | 0.00999021 | 0.00999021 | -3.9947576 | 2.57616786 |

**Supplemental Table S4: Differentially Expressed Genes (DEGs) from Vehicle Control after Reelin (50 nᴍ)**

| **gene_name** | **logFC** | **AveExpr** | **t** | **P.Value** | **adj.P.Val** | **B** | **z.std** |
| --- | --- | --- | --- | --- | --- | --- | --- |
| *ATP5MC2P4* | 4.93837161 | -5.3933622 | 5.42999775 | 3.0133E-05 | 3.0133E-05 | -3.1226152 | 4.17245608 |
| *MIR27B* | -4.6611904 | -5.2522459 | -5.5718877 | 4.8875E-05 | 4.8875E-05 | -3.4923351 | -4.0609447 |
| *PSPHP1* | -0.5639907 | -3.1163877 | -4.7189231 | 0.00026898 | 0.00026898 | -3.6933951 | -3.643481 |
| *IQCD* | -0.6255779 | 2.35314126 | -4.7155858 | 0.00026988 | 0.00026988 | -3.5967485 | -3.6426151 |
| *TMEM44-AS1* | -0.7304377 | 2.43113552 | -4.620193 | 0.00031881 | 0.00031881 | -3.8094764 | -3.5995173 |
| *GPR52* | -5.3016871 | -5.323325 | -4.3665884 | 0.00032822 | 0.00032822 | -3.5470224 | -3.591944 |
| *PRR15-DT* | 4.29866774 | -5.2530842 | 4.43026694 | 0.00040239 | 0.00040239 | -3.5376427 | 3.53850999 |
| *ONECUT3* | 3.77806629 | -5.9035052 | 4.29047726 | 0.000576 | 0.000576 | -3.5730814 | 3.44267008 |
| *WFIKKN1* | -0.5374531 | 3.04489781 | -4.2293602 | 0.00071909 | 0.00071909 | -3.8117258 | -3.3821941 |
| *GGCTP1* | 3.98258238 | -5.4604676 | 4.20957725 | 0.00074294 | 0.00074294 | -3.671806 | 3.37322057 |
| *LMOD1* | 0.95029657 | 0.98687926 | 4.16523578 | 0.00081049 | 0.00081049 | -3.7745933 | 3.34918733 |
| *HMGB1P14* | -4.8936742 | -4.7762225 | -4.1251879 | 0.00085233 | 0.00085233 | -3.7866745 | -3.3352153 |
| *RNF207-AS1* | -5.0529628 | -3.7916028 | -4.1270085 | 0.00086033 | 0.00086033 | -3.8964629 | -3.3326169 |
| *C9orf152* | 3.76243837 | -5.2799363 | 4.05515121 | 0.00089933 | 0.00089933 | -3.6399429 | 3.32026282 |
| *CHRND* | -6.4029547 | -4.7568679 | -4.1230068 | 0.00093731 | 0.00093731 | -3.8099825 | -3.308699 |
| *ORMDL1P1* | -4.1641419 | -5.8573853 | -3.8174781 | 0.00115292 | 0.00115292 | -3.6698038 | -3.2502796 |
| *OSER1* | -0.2885048 | 5.5465404 | -3.9866212 | 0.00117579 | 0.00117579 | -3.7906235 | -3.2446897 |
| *ALOXE3P1* | -3.8440723 | -5.925008 | -3.9604794 | 0.00123398 | 0.00123398 | -3.7541245 | -3.2309082 |
| *LINC01585* | -4.714321 | -5.5840476 | -4.0937131 | 0.00125345 | 0.00125345 | -3.6941506 | -3.2264292 |
| *MYCNOS* | -1.14135 | 2.50624193 | -3.9371812 | 0.00127617 | 0.00127617 | -3.8354083 | -3.221286 |
| *MGC32805* | 4.56672539 | -5.350645 | 3.85208253 | 0.00141097 | 0.00141097 | -3.6729394 | 3.19239668 |
| *OVOL2* | 4.70682592 | -5.0378256 | 3.72819107 | 0.00141412 | 0.00141412 | -3.7033358 | 3.19175251 |
| *TAS2R43* | 4.01432494 | -5.5271401 | 3.71273044 | 0.00146499 | 0.00146499 | -3.6493054 | 3.18153099 |
| *PPIAP42* | -4.5198685 | -5.0661646 | -3.6868421 | 0.00155426 | 0.00155426 | -3.7574182 | -3.1643574 |
| *HOXC10* | -1.4094987 | -5.8136243 | -3.7780893 | 0.00179113 | 0.00179113 | -3.8246552 | -3.122844 |
| *BTBD19* | 0.80274714 | 2.24361231 | 3.72187667 | 0.00203647 | 0.00203647 | -3.8489735 | 3.08486215 |
| *MORF4* | 4.29560107 | -4.4054958 | 3.53437767 | 0.0022006 | 0.0022006 | -3.7446209 | 3.06173243 |
| *ZNF436-AS1* | -0.7716472 | 2.32310819 | -3.6703567 | 0.00222281 | 0.00222281 | -3.9302685 | -3.0587254 |
| *NPIPB15* | -0.9093964 | 2.71727257 | -3.6234537 | 0.00250597 | 0.00250597 | -4.0668525 | -3.0226192 |
| *FTH1P10* | 3.69109988 | -4.2945613 | 3.64714239 | 0.00253464 | 0.00253464 | -3.8950393 | 3.01917482 |
| *FUT5* | 4.45942392 | -4.8812646 | 3.57547991 | 0.00265023 | 0.00265023 | -3.818139 | 3.00563934 |
| *SLC2A14* | 2.90976334 | -2.5949959 | 3.55862598 | 0.00269549 | 0.00269549 | -3.8414805 | 3.00048634 |
| *SLC7A5* | 0.48272929 | 7.12866452 | 3.55808754 | 0.00283372 | 0.00283372 | -3.8563746 | 2.98522316 |
| *FRG1EP* | 3.753666 | -4.7997885 | 3.54081952 | 0.0030109 | 0.0030109 | -3.8651494 | 2.96662304 |
| *HLA-DRB5* | 0.86743028 | -1.9537274 | 3.49657014 | 0.0032079 | 0.0032079 | -3.7863079 | 2.94708008 |
| *MRPL37P1* | -4.873272 | -3.3756978 | -3.4473502 | 0.00330743 | 0.00330743 | -4.0152052 | -2.9376191 |
| *ANKRD12* | 0.29269593 | 5.38343515 | 3.46612249 | 0.0034238 | 0.0034238 | -3.9195402 | 2.92688048 |
| *CPEB1* | -0.4409411 | 1.90768021 | -3.454718 | 0.00351485 | 0.00351485 | -3.965357 | -2.9187082 |
| *PPP1R18* | 0.23715117 | 6.31044094 | 3.42507327 | 0.00372525 | 0.00372525 | -3.9191 | 2.90053625 |
| *ETS2* | 0.47305226 | 3.84280857 | 3.38643309 | 0.00401749 | 0.00401749 | -3.9058842 | 2.8767848 |
| *PTOV1P1* | 4.73914872 | -4.8640602 | 3.26593101 | 0.00404333 | 0.00404333 | -3.852448 | 2.87476157 |
| *HOATZ* | -0.7009038 | 2.15800685 | -3.363628 | 0.00426173 | 0.00426173 | -3.8808779 | -2.8581099 |
| *OGFR-AS1* | -3.1008516 | -3.3777216 | -3.2338269 | 0.00434647 | 0.00434647 | -3.996106 | -2.8518568 |
| *LINC02703* | 4.30516899 | -4.8451875 | 3.30851 | 0.00447144 | 0.00447144 | -3.8639937 | 2.84283348 |
| *TLK2P2* | -4.1939351 | -4.9201442 | -3.3670054 | 0.00450427 | 0.00450427 | -3.9637898 | -2.8405014 |
| *MYPN* | -4.1196809 | -5.8463109 | -3.3306827 | 0.00456851 | 0.00456851 | -3.9139643 | -2.8359812 |
| *DLX4* | 2.41967569 | -5.3118304 | 3.31258328 | 0.00457136 | 0.00457136 | -3.9105372 | 2.83578254 |
| *ACOX2* | -0.6866108 | 2.41890207 | -3.30071 | 0.00486064 | 0.00486064 | -3.9611326 | -2.8161272 |
| *LNX2* | 0.42696511 | 2.97790156 | 3.28638779 | 0.00491482 | 0.00491482 | -3.9273154 | 2.81256448 |
| *RNU6-247P* | -2.7575153 | -4.2450068 | -3.2859748 | 0.00493078 | 0.00493078 | -4.0470766 | -2.8115217 |
| *HMGN5* | -0.3433929 | 3.97980641 | -3.2713472 | 0.00500279 | 0.00500279 | -3.9765007 | -2.8068543 |
| *GPD2* | 0.40832928 | 3.55494137 | 3.27839939 | 0.00501914 | 0.00501914 | -3.9415666 | 2.80580266 |
| *LRRC74B* | -0.611686 | 3.07003941 | -3.2656661 | 0.00518457 | 0.00518457 | -3.9847876 | -2.7953367 |
| *TMEM232* | -0.5951495 | 2.59605838 | -3.2532493 | 0.00530665 | 0.00530665 | -3.9634118 | -2.7878049 |
| *ATP6V0C* | 2.9961468 | -2.3242885 | 3.24310941 | 0.00538551 | 0.00538551 | -4.0373119 | 2.78302225 |
| *DLX3* | 4.05173873 | -3.6391594 | 3.18867289 | 0.00560784 | 0.00560784 | -3.964415 | 2.76987168 |
| *LINC02104* | 3.77517876 | -5.758445 | 3.19468759 | 0.0056778 | 0.0056778 | -3.9048126 | 2.76583058 |
| *TNNT3* | -4.5219712 | -5.3550568 | -3.2019305 | 0.00572876 | 0.00572876 | -3.9613117 | -2.7629158 |
| *CSNK1G2-AS1* | 4.79718933 | -4.0398953 | 3.15665379 | 0.00575272 | 0.00575272 | -3.9368285 | 2.76155273 |
| *PLA2G12AP1* | -3.2908225 | -1.9631829 | -3.212328 | 0.0058108 | 0.0058108 | -4.1628751 | -2.758271 |
| *EEF1A1P24* | 3.48246411 | -4.8832371 | 3.09139299 | 0.0059811 | 0.0059811 | -3.9497854 | 2.74881558 |
| *DNAJC8P4* | -2.5624492 | -5.5620186 | -3.1684002 | 0.00608516 | 0.00608516 | -3.8757647 | -2.7431569 |
| *UBL5P2* | -4.5056485 | -4.3091187 | -3.1710147 | 0.00632216 | 0.00632216 | -4.0718769 | -2.7305872 |
| *KCNJ11* | -0.5769563 | 1.82773561 | -3.1740079 | 0.00633053 | 0.00633053 | -4.0130034 | -2.7301514 |
| *BST1* | 0.38072044 | 2.93801989 | 3.15943411 | 0.00642603 | 0.00642603 | -3.9228407 | 2.7252118 |
| *FGF2* | 0.66551608 | 3.19399411 | 3.15826758 | 0.00644782 | 0.00644782 | -3.9577149 | 2.7240938 |
| *SLC4A9* | 4.36027242 | -4.7996772 | 3.04904321 | 0.00657317 | 0.00657317 | -3.9204427 | 2.71772874 |
| *CACNA1F* | -3.3615241 | -5.8390916 | -3.1192138 | 0.00662088 | 0.00662088 | -3.9459958 | -2.7153346 |
| *C6orf132* | 3.50150061 | -3.7754419 | 3.03706892 | 0.00675064 | 0.00675064 | -3.9443131 | 2.70890148 |
| *OR52N4* | -2.7002949 | -4.6100886 | -3.1153537 | 0.00693848 | 0.00693848 | -4.0230977 | -2.6997826 |
| *OTOS* | -0.7044932 | 1.97217351 | -3.1226455 | 0.00693865 | 0.00693865 | -4.0118765 | -2.6997743 |
| *IL12RB2* | 3.76497728 | -4.0884541 | 3.02195652 | 0.00698125 | 0.00698125 | -3.9037433 | 2.69773721 |
| *FOSL2-AS1* | -2.8292817 | -4.5000861 | -3.1134662 | 0.00702115 | 0.00702115 | -4.0409566 | -2.6958394 |
| *PPIAL4D* | -3.583118 | -3.003096 | -3.0004944 | 0.00732192 | 0.00732192 | -4.0854804 | -2.6818367 |
| *TTC39C* | 0.39167215 | 3.87428446 | 3.08958932 | 0.0073624 | 0.0073624 | -4.0258285 | 2.67999175 |
| *COPB2-DT* | -0.4492861 | 3.19847409 | -3.0708337 | 0.00768199 | 0.00768199 | -3.989894 | -2.6657362 |
| *EBI3* | 4.61163993 | -4.6470675 | 3.04860617 | 0.00777493 | 0.00777493 | -4.0347519 | 2.66169046 |
| *ADAMTS9-AS1* | 3.50089609 | -2.8488636 | 3.08029012 | 0.00796383 | 0.00796383 | -4.0583229 | 2.65359959 |
| *TCP1P1* | 3.0806242 | -5.6012634 | 3.02876314 | 0.00804543 | 0.00804543 | -4.0096592 | 2.65015737 |
| *SCRG1* | 0.3368394 | 4.93064804 | 3.03860095 | 0.00823761 | 0.00823761 | -4.018092 | 2.64217263 |
| *MAILR* | -0.3450635 | 4.12649818 | -3.0302168 | 0.00834044 | 0.00834044 | -4.0364287 | -2.6379683 |
| *RPL23AP81* | -2.5336899 | -4.2469773 | -3.0202069 | 0.00848054 | 0.00848054 | -4.0435004 | -2.6323141 |
| *HERC2P6* | 0.84839637 | -5.7901317 | 3.01713448 | 0.00859063 | 0.00859063 | -4.0195446 | 2.62792948 |
| *LMNTD1* | -1.4896998 | -5.8616685 | -3.0134449 | 0.00861828 | 0.00861828 | -4.0236425 | -2.626836 |
| *COL8A2* | 0.67121542 | 2.07241313 | 3.01350527 | 0.00866806 | 0.00866806 | -4.0488184 | 2.62487579 |
| *C8orf89* | 3.8989161 | -3.3015447 | 2.9933126 | 0.00867314 | 0.00867314 | -4.0520632 | 2.62467629 |
| *PSMD14-DT* | -4.3260581 | -5.2932543 | -3.0635664 | 0.00876983 | 0.00876983 | -4.0003028 | -2.6208988 |
| *RPS10P9* | 3.57220151 | -5.7276955 | 2.91881409 | 0.00877148 | 0.00877148 | -3.9730806 | 2.62083463 |
| *ARHGAP8* | -4.9037062 | -3.6216071 | -2.9817339 | 0.00900082 | 0.00900082 | -4.0758556 | -2.6120231 |
| *H19* | -0.6987618 | -0.9213935 | -2.9938281 | 0.00904042 | 0.00904042 | -3.9930753 | -2.610522 |
| *RPS4XP6* | 3.28004271 | -3.6982685 | 2.96753787 | 0.00917999 | 0.00917999 | -4.0291023 | 2.6052774 |
| *DCDC2* | -0.410825 | 3.1739588 | -2.9852978 | 0.00918489 | 0.00918489 | -4.0472882 | -2.6050945 |
| *SLC25A31* | 3.79394386 | -5.28529 | 2.89740327 | 0.00919504 | 0.00919504 | -3.9392613 | 2.60471609 |
| *CENPP* | -0.5832757 | 1.74325402 | -2.9645777 | 0.00963356 | 0.00963356 | -4.0916333 | -2.5887122 |
| *LINC01797* | -3.9274968 | -4.0713543 | -2.9623837 | 0.00967338 | 0.00967338 | -4.1161537 | -2.5872911 |
| *NPM1P43* | 3.74850788 | -4.9646593 | 2.87145965 | 0.00973469 | 0.00973469 | -4.01611 | 2.58511376 |
| *FAM72B* | 1.04529822 | 0.3888231 | 2.95371026 | 0.00980013 | 0.00980013 | -4.0403951 | 2.58280271 |
| *PTMAP4* | 2.12162779 | -2.461143 | 2.86627771 | 0.00984607 | 0.00984607 | -4.0716585 | 2.58118904 |

**Supplemental Table S5: Differentially Expressed Genes (DEGs) from Vehicle Control after (2*R*,6*R*)-Hydroxynorketamine (1 µᴍ)**

| **gene_name** | **logFC** | **AveExpr** | **t** | **P.Value** | **adj.P.Val** | **B** | **z.std** |
| --- | --- | --- | --- | --- | --- | --- | --- |
| *PSPHP1* | -0.6083259 | -3.1163877 | -5.6505463 | 4.4838E-05 | 4.4838E-05 | -3.3365142 | -4.0810251 |
| *DNAJC8P4* | -4.5197673 | -5.5620186 | -5.5997728 | 4.8349E-05 | 4.8349E-05 | -3.190454 | -4.0634695 |
| *IL17REL* | 4.29146519 | -5.8470161 | 4.79075365 | 0.00022869 | 0.00022869 | -3.3464001 | 3.68501923 |
| *HDAC8* | -0.5876476 | 3.63692049 | -4.6723667 | 0.00029401 | 0.00029401 | -3.5542924 | -3.6205221 |
| *PPIAP42* | -4.8954157 | -5.0661646 | -4.3193918 | 0.00036556 | 0.00036556 | -3.4247323 | -3.5637744 |
| *CACNA1F* | -4.3531525 | -5.8390916 | -4.3487712 | 0.00053661 | 0.00053661 | -3.449505 | -3.4617817 |
| *RPSAP54* | 2.08335987 | -1.4715367 | 4.32049129 | 0.00059938 | 0.00059938 | -3.6889653 | 3.43189328 |
| *IRX6* | -1.3267802 | -3.6665557 | -4.3028749 | 0.00061735 | 0.00061735 | -3.7268576 | -3.4238759 |
| *GGCTP1* | 3.73823557 | -5.4604676 | 4.07253831 | 0.00103738 | 0.00103738 | -3.6605623 | 3.28018682 |
| *MIR27B* | -3.0212666 | -5.2522459 | -4.0078603 | 0.00109549 | 0.00109549 | -3.6458707 | -3.2647791 |
| *CFAP97D1* | 3.77007271 | -3.1562764 | 3.95271916 | 0.00120788 | 0.00120788 | -3.808921 | 3.23701242 |
| *TNFRSF17* | 4.1989538 | -4.1186305 | 3.7475196 | 0.001353 | 0.001353 | -3.7090772 | 3.20449491 |
| *TCP1P1* | 3.63674396 | -5.6012634 | 3.8649807 | 0.00146752 | 0.00146752 | -3.6499008 | 3.1810301 |
| *LINC02350* | 4.48973616 | -4.8973091 | 3.87609347 | 0.00148211 | 0.00148211 | -3.6753785 | 3.17816328 |
| *RPL7P37* | 2.96260152 | -5.8495101 | 3.69562929 | 0.00152337 | 0.00152337 | -3.6139725 | 3.17019473 |
| *ORMDL1P1* | -3.692559 | -5.8573853 | -3.6452659 | 0.00170905 | 0.00170905 | -3.619448 | -3.1366245 |
| *INTS4P2* | 2.91858168 | -5.0658479 | 3.77700941 | 0.00177234 | 0.00177234 | -3.6353343 | 3.12594589 |
| *ACKR4* | 3.64978069 | -3.6525838 | 3.78837623 | 0.00179314 | 0.00179314 | -3.7968578 | 3.1225133 |
| *GNG5B* | 3.43139991 | -4.8592238 | 3.62165574 | 0.00180366 | 0.00180366 | -3.6217215 | 3.12079176 |
| *MIR194-2HG* | 3.93179343 | -5.4549992 | 3.58885059 | 0.00194377 | 0.00194377 | -3.6660043 | 3.0986915 |
| *NUDCP2* | 3.75115136 | -3.5302708 | 3.56615404 | 0.00204697 | 0.00204697 | -3.7937536 | 3.083332 |
| *LCN9* | -4.0004804 | -3.6166041 | -3.6749592 | 0.00224987 | 0.00224987 | -3.9112385 | -3.0550984 |
| *KCNIP1-AS1* | 4.16783466 | -5.1760543 | 3.64394695 | 0.00231146 | 0.00231146 | -3.7782496 | 3.04698898 |
| *LINC00595* | 0.58011081 | 1.46638699 | 3.59772996 | 0.002603 | 0.002603 | -3.7657444 | 3.01110345 |
| *H19* | -0.9912987 | -0.9213935 | -3.5720125 | 0.00275942 | 0.00275942 | -4.0076502 | -2.9933403 |
| *MYL11* | -4.0117645 | -2.1198256 | -3.5024454 | 0.00292663 | 0.00292663 | -3.6427746 | -2.9753415 |
| *FAM162B* | 3.80621618 | -5.7756325 | 3.58098547 | 0.00309692 | 0.00309692 | -3.6745767 | 2.95795074 |
| *RPAP3* | 0.39177846 | 4.43704285 | 3.50117998 | 0.00321105 | 0.00321105 | -3.8135107 | 2.94677658 |
| *DMRT3* | 1.3353997 | -0.371635 | 3.49710168 | 0.00324619 | 0.00324619 | -3.6791914 | 2.9434097 |
| *NACA4P* | 3.4554915 | -3.9231729 | 3.3622388 | 0.00325289 | 0.00325289 | -3.8549394 | 2.94277096 |
| *CACNA2D3* | 0.50276539 | 3.68899774 | 3.44677869 | 0.00357544 | 0.00357544 | -3.844714 | 2.91337572 |
| *SMARCAL1-AS1* | 3.6159846 | -5.2908695 | 3.44467486 | 0.00361557 | 0.00361557 | -3.7689899 | 2.9098887 |
| *GAPDHP44* | -2.8969522 | -5.75069 | -3.4026544 | 0.00387869 | 0.00387869 | -3.8500013 | -2.8878625 |
| *H2AC4* | 3.27613486 | -5.5193097 | 3.3907061 | 0.00407215 | 0.00407215 | -3.8146096 | 2.87251797 |
| *SUB1P1* | 4.70932924 | -4.8535479 | 3.25423901 | 0.00415125 | 0.00415125 | -3.8414513 | 2.86643346 |
| *FOXC1* | 0.92136918 | 1.24724926 | 3.39464081 | 0.00416925 | 0.00416925 | -3.8298463 | 2.86506418 |
| *HSPA8P9* | -3.2985368 | -5.4626392 | -3.3667533 | 0.00420827 | 0.00420827 | -3.8630619 | -2.8621128 |
| *ENTPD5* | 0.70310155 | 2.57107055 | 3.38447697 | 0.00422397 | 0.00422397 | -3.8231822 | 2.86093248 |
| *SLC2A3P4* | -3.3954073 | -5.8506468 | -3.3640212 | 0.00426087 | 0.00426087 | -3.8305855 | -2.8581738 |
| *STAT4-AS1* | -3.7186961 | -5.4186681 | -3.3410698 | 0.00452432 | 0.00452432 | -3.8398112 | -2.8390845 |
| *DGKZP1* | 1.08985663 | -1.7708913 | 3.32609856 | 0.00453032 | 0.00453032 | -3.8955571 | 2.83866146 |
| *SEL1L2* | 3.36234986 | -6.0096073 | 3.21143446 | 0.00457095 | 0.00457095 | -3.7972449 | 2.83581125 |
| *RPL4P6* | 3.77779376 | -4.6195223 | 3.21009788 | 0.0045847 | 0.0045847 | -3.844506 | 2.8348517 |
| *AMMECR1L* | 0.30804239 | 5.24641339 | 3.31776239 | 0.00465213 | 0.00465213 | -3.8771714 | 2.83018367 |
| *TPH2* | 2.88719807 | -5.7862712 | 3.32325253 | 0.00470976 | 0.00470976 | -3.8034741 | 2.82624249 |
| *SBSPON* | 0.64176974 | 2.48204233 | 3.31133155 | 0.00473668 | 0.00473668 | -3.9174656 | 2.82441662 |
| *SNORA31* | -3.9811089 | -4.567629 | -3.1658523 | 0.00506341 | 0.00506341 | -3.8623291 | -2.8029719 |
| *HAS2* | 0.77129205 | 2.72821312 | 3.27599042 | 0.00514054 | 0.00514054 | -3.8419574 | 2.7980922 |
| *HOXB3* | 3.39364335 | -3.1487424 | 3.25913885 | 0.00533945 | 0.00533945 | -4.1291515 | 2.78580817 |
| *RPL10P9* | -0.6131429 | 2.12167166 | -3.2392335 | 0.00544108 | 0.00544108 | -3.7814679 | -2.7796899 |
| *CPSF4L* | -2.4077354 | -3.9829147 | -3.2301218 | 0.00555306 | 0.00555306 | -3.9119836 | -2.7730675 |
| *ODC1-DT* | 0.6816918 | 1.14625388 | 3.21544253 | 0.00557438 | 0.00557438 | -3.8936028 | 2.77182025 |
| *MRPS5* | 0.55689348 | 1.50377469 | 3.22298134 | 0.00567137 | 0.00567137 | -3.9285776 | 2.76620005 |
| *BANF1P1* | 3.43186522 | -5.9818629 | 3.2144696 | 0.00567159 | 0.00567159 | -3.865668 | 2.76618768 |
| *POF1B* | 3.36987309 | -5.0481555 | 3.1967195 | 0.00581043 | 0.00581043 | -3.9337149 | 2.75829206 |
| *TIPARP* | 0.50576734 | 4.88190311 | 3.20431264 | 0.00586376 | 0.00586376 | -3.8906428 | 2.75530431 |
| *MRPS9* | -0.373865 | 3.41304938 | -3.1991379 | 0.00599795 | 0.00599795 | -3.8748652 | -2.7478934 |
| *TNNT3* | -4.1310498 | -5.3550568 | -3.1478777 | 0.00643034 | 0.00643034 | -3.9009145 | -2.7249902 |
| *PPAT* | 0.44550806 | 3.82561902 | 3.16867476 | 0.00652214 | 0.00652214 | -3.9063777 | 2.72030654 |
| *EEF1A1P24* | 3.30008543 | -4.8832371 | 3.03223355 | 0.00682361 | 0.00682361 | -3.9346664 | 2.70533223 |
| *MIR4500HG* | 0.66956514 | 2.13012683 | 3.09148986 | 0.00741239 | 0.00741239 | -4.0306604 | 2.67772555 |
| *HTR3B* | 3.59039409 | -3.2604651 | 3.06744682 | 0.00742814 | 0.00742814 | -4.0740326 | 2.67701453 |
| *SYTL2* | 0.41545239 | 4.02686479 | 3.08854164 | 0.00743305 | 0.00743305 | -3.9574671 | 2.67679302 |
| *LINC02188* | -4.16444 | -3.8652406 | -3.0783856 | 0.00746937 | 0.00746937 | -3.9931625 | -2.6751593 |
| *RPL7AP2* | -3.2058673 | -5.9597607 | -3.0984842 | 0.00760209 | 0.00760209 | -3.9026988 | -2.6692499 |
| *RAF1* | 0.60414363 | 3.79175227 | 3.02361552 | 0.00825196 | 0.00825196 | -3.9466259 | 2.641583 |
| *CLASP1-AS1* | -3.3987163 | -1.9536363 | -2.9412035 | 0.00834869 | 0.00834869 | -4.0884601 | -2.6376329 |
| *SGCA* | -4.7355607 | -3.7499698 | -3.0215578 | 0.00835726 | 0.00835726 | -4.0645557 | -2.6372849 |
| *GPR183* | -3.4667275 | -4.0811579 | -3.0034504 | 0.00869481 | 0.00869481 | -4.0471798 | -2.6238263 |
| *SNHG16* | 0.23706191 | 4.11355798 | 3.01205152 | 0.00871441 | 0.00871441 | -3.9782406 | 2.62305913 |
| *GPR52* | -3.0586035 | -5.323325 | -2.9163886 | 0.0088185 | 0.0088185 | -3.8658368 | -2.6190113 |
| *PACRG-AS3* | 4.174898 | -3.6733786 | 2.91498076 | 0.00884591 | 0.00884591 | -4.0080313 | 2.61795273 |
| *CD177* | 2.01153637 | -5.3051469 | 2.99330965 | 0.00891218 | 0.00891218 | -3.9662199 | 2.61540463 |
| *ZNF33A* | 0.26330811 | 5.25496711 | 3.00014579 | 0.00891747 | 0.00891747 | -3.9667658 | 2.61520213 |
| *RSL24D1P1* | 4.07974103 | -5.2407208 | 2.97206433 | 0.00930222 | 0.00930222 | -3.9413506 | 2.60074276 |
| *MIRLET7IHG* | 0.4740372 | 3.87063948 | 2.97445332 | 0.00945896 | 0.00945896 | -3.9781179 | 2.59500466 |
| *RPSAP9* | -1.0335112 | 0.67923341 | -2.9627933 | 0.00968321 | 0.00968321 | -4.0403365 | -2.5869411 |
| *OR52N4* | -2.4082849 | -4.6100886 | -2.9487926 | 0.00980149 | 0.00980149 | -4.0176782 | -2.5827548 |
| *BRWD1-AS1* | 3.67242057 | -4.0675511 | 2.86716928 | 0.00982682 | 0.00982682 | -3.970306 | 2.58186452 |
| *MMAA* | 0.82789803 | 2.68773397 | 2.92287106 | 0.00992751 | 0.00992751 | -4.0777399 | 2.57834397 |
| *CNKSR3* | -0.4440008 | 2.91859795 | -2.9541524 | 0.00998121 | 0.00998121 | -3.9662428 | -2.5764797 |
